# Parkinson’s disease determinants, prediction and gene–environment interactions in the UK Biobank

**DOI:** 10.1136/jnnp-2020-323646

**Published:** 2020-09-14

**Authors:** Benjamin Meir Jacobs, Daniel Belete, Jonathan Bestwick, Cornelis Blauwendraat, Sara Bandres-Ciga, Karl Heilbron, Ruth Dobson, Mike A Nalls, Andrew Singleton, John Hardy, Gavin Giovannoni, Andrew John Lees, Anette-Eleonore Schrag, Alastair J Noyce

**Affiliations:** 1 Preventive Neurology Unit, Wolfson Institute of Preventive Medicine, Barts and The London School of Medicine and Dentistry, London, UK; 2 Laboratory of Neurogenetics, National Institute on Aging, National Institutes of Health, Bethesda, Maryland, USA; 3 23andMe, Mountain View, California, USA; 4 Department of Molecular Neuroscience, UCL Institute of Neurology, London, UK; 5 Centre for Neuroscience and Trauma, Barts and The London School of Medicine and Dentistry, Blizard Institute, London, UK; 6 Reta Lila Weston Institute of Neurological Studies and Department of Clinical and Movement Neurosciences, UCL Institute of Neurology, London, UK; 7 Department of Clinical and Movement Neurosciences, UCL Institute of Neurology, London, UK

## Abstract

**Objective:**

To systematically investigate the association of environmental risk factors and prodromal features with incident Parkinson’s disease (PD) diagnosis and the interaction of genetic risk with these factors. To evaluate whether existing risk prediction algorithms are improved by the inclusion of genetic risk scores.

**Methods:**

We identified individuals with an incident diagnosis of PD (n=1276) and controls (n=500 406) in UK Biobank. We determined the association of risk factors with incident PD using adjusted logistic regression models. We constructed polygenic risk scores (PRSs) using external weights and selected the best PRS from a subset of the cohort (30%). The PRS was used in a separate testing set (70%) to examine gene–environment interactions and compare predictive models for PD.

**Results:**

Strong evidence of association (false discovery rate <0.05) was found between PD and a positive family history of PD, a positive family history of dementia, non-smoking, low alcohol consumption, depression, daytime somnolence, epilepsy and earlier menarche. Individuals with the highest 10% of PRSs had increased risk of PD (OR 3.37, 95% CI 2.41 to 4.70) compared with the lowest risk decile. A higher PRS was associated with earlier age at PD diagnosis and inclusion of the PRS in the PREDICT-PD algorithm led to a modest improvement in model performance. We found evidence of an interaction between the PRS and diabetes.

**Interpretation:**

Here, we used UK Biobank data to reproduce several well-known associations with PD, to demonstrate the validity of a PRS and to demonstrate a novel gene–environment interaction, whereby the effect of diabetes on PD risk appears to depend on background genetic risk for PD.

## Introduction

Parkinson’s disease (PD) is the second most prevalent neurodegenerative disorder worldwide.^1^ By the time an individual is diagnosed with PD, a substantial proportion of nigrostriatal neurons has already been lost.^2^ Identification of at-risk individuals and earlier detection likely represent the best opportunities for the development of effective treatments to prevent or reverse progression of PD.

Over the past decade, large genome-wide association studies (GWAS) of PD have built on linkage studies of rare, familial forms of PD. From the latest PD GWAS, 90 independent signals were identified, which collectively explain ~16% of overall PD liability.^3^ Separately, epidemiological studies have identified potentially modifiable exposures, various comorbidities and prodromal features.^4 5^ There have been efforts to incorporate these non-genetic risk factors into predictive algorithms to identify individuals at higher risk of PD.^6–9^

The modest overall liability explained by genetic factors and small individual effect sizes of environmental risk factors for PD suggest that interactions between them may explain some of the missing risk. Modelling interactions may yield insights into PD pathobiology, further improve prediction algorithms and suggest potential ways to modify risk through intervention in geneticallystratified groups.^10–12^

Here, we used the UK Biobank (UKB) cohort and the latest PD GWAS data to evaluate the association of environmental and prodromal factors with incident PD, explore how a polygenic risk score (PRS) for PD improves the performance of a prediction algorithm that combines these factors and explore how the PRS interacts with environmental/prodromal factors.

## Methods

### Data sources

The UKB is a large repository that contains health-related data on over 500 000 individuals across the UK. The methods by which these data were collected have been described elsewhere.^13^ Briefly, between 2006 and 2010 adults aged between 40 and 69 years within close proximity to 1 of 22 UKB recruitment centres were invited to participate. Individuals had extensive demographic, lifestyle, clinical and radiological information collected. In addition to this, participants underwent genotyping and had health records collected using linked Hospital Episode Statistics.

### Study design, definition of exposures and outcomes

For the analyses assessing the association of environmental and prodromal factors, we included only incident cases of PD (those individuals in whom the diagnosis was recorded after their UKB initial assessment visit) and excluded prevalent cases (individuals diagnosed with PD prior to their initial assessment visit). PD diagnoses were derived from self-report or linked Hospital Episode Statistics ICD codes (online supplementary table 1). We included all participants without a PD diagnosis in the dataset as unmatched controls and adjusted for relevant confounding factors in the subsequent analyses. Of note, age at completion of full-time education was only available for a subset of participants (n=330 240). We chose to control for deprivation, a useful proxy for socioeconomic status, in our models to prevent exclusion of the ~170 000 individuals with missing education data.

10.1136/jnnp-2020-323646.supp1 Supplementary data

We attempted to include all exposures shown to be associated with PD risk in a large meta-analysis.^4^ All exposures were captured at the time of the initial visit. Details of how each exposure variable was defined are provided in online supplementary table 1. Exposures were excluded from this analysis if the reported prevalence in UKB was substantially lower than reported population prevalence (i.e. anosmia, erectile dysfunction, shoulder pain/stiffness)^14–18^ and therefore deemed unreliably recorded.

### Genotype data

Genotyping was performed using the Axiom (UK Biobank Axiom Array, ThermoFisher) and UK BiLEVE arrays. Genotyping, imputation and quality control procedures are described elsewhere.^19^ Genetic principal components were supplied by UKB (data-field 22 009).

### Construction of PRS

A variety of PRS were created using the clumping-and-thresholding approach:

1. We extracted variant associations with PD from the most recent GWAS but not including the UKB participants from that GWAS.^3^
2. We excluded palindromic variants and variants without an rsID.
3. We excluded variants associated with PD above an arbitrary p value threshold (0.00005, 0.0005, 0.005, 0.05, 0.1, 0.2, 0.4, 0.6, 0.8, and 1).
4. We clumped using several r^2^ thresholds (0.1, 0.2, 0.4, 0.6, 0.8) and a clumping distance of 250 kb, with the 1000 genomes EUR samples as the reference genome.^20^

Reference genome data were obtained from the 503 participants of European ancestry in the 1000 genomes project.^21^ Only autosomal, biallelic variants which passed quality control in both the PD GWAS and target (UKB) datasets were included. We excluded all duplicate rsIDs, duplicate positions, variants deviating from Hardy-Weinberg equilibrium (p<1e-06), rare variants with minor allele frequencies <0.01, variants with genotype missingness >10% and variants with low imputation quality (Mach R^2^ <0.3). After SNP QC, a total of 4 490 455 markers overlapped between the reference and target datasets. For genetic analysis, individuals with >10% missing genotypes were excluded, and only individuals with self-reported ‘White British’ ethnicity and genetically European ancestry as defined by genetic principal components were included. We excluded one of each pair of individuals related at a kinship coefficient cut-off of 0.0442, equivalent to a third-degree relative. Kinship coefficients were calculated by UKB and are provided in the ‘Relatedness’ file (category 263).

As a sensitivity analysis to determine whether as-yet-undiscovered genomic risk loci explained additional liability to PD, we created an additional PRS using the best-performing PRS (in terms of Nagelkerke’s pseudo-R^2^). We excluded all variants within 1 MB either side of the lead SNP for the 90 risk loci discovered in the most recent International Parkinson’s Disease Genomics Consortium (IPDGC) GWAS.^3^

Effect allele dosage at each locus was multiplied by the beta coefficient to generate the risk score for that locus. Scores were standardised to have mean 0 and a 1-unit variance for each SNP. For missing genotypes, the score at that locus was defined as the mean of all scores at that locus. Risk scores were totalled across the genome to calculate an individual’s score. All individuals in UKB with a PD diagnosis, prevalent or incident, were included. Analysis was performed in PLINK (V.2.00aLM 64-bit Intel) using the ‘--score’ flag.

### Statistical methods

#### Incident case–control study

Multivariable logistic regression models were built for each risk factor using the entire UKB cohort as controls and adjusting for age, sex, ethnicity and deprivation status. Models were of the form: PD status ~Age+Sex+Ethnicity+Townsend Deprivation status+risk factor. Next, a multivariable logistic regression model was built for incident PD comprising all environmental factors robustly associated (false discovery rate (FDR) <0.05) with PD risk, including the above confounders. Likelihood ratio tests were used to assess the improvement of model fit at an FDR threshold of 0.05, that is, for each risk factor the above model was compared with a null model of the form: PD status ~Age+Sex+Ethnicity+Townsend Deprivation status. For sex-specific covariates, sex was not included as a confounding covariate. We performed the following sensitivity analyses: exclusion of individuals under the age of 60, exclusion of non-White individuals, exclusion of individuals whose PD diagnosis was solely derived from self-report and a matched case–control analysis (each case matched to exactly four participants for age, ethnicity and sex).

#### Application of the PREDICT-PD algorithm

We applied the PREDICT-PD algorithm to UKB participants to externally validate this risk score and determine whether its predictive performance was enhanced by the addition of a genetic risk score.^7^ The algorithm uses published estimates of relative risks and ORs derived from large meta-analyses of early non-motor features and risk factors for PD.^4^ Baseline risk of PD (on the odds scale) was determined from the following equation^7^:

$$odds\left(PD\right)=\frac{Pr\left(PD\right)}{1-Pr\left(PD\right)}=\frac{1}{1+28.53049+73.67057\times exp(-0.165308(Age-60))}$$

With the PREDICT-PD algorithm, the following adjustments to this baseline age-adjusted risk are made for individuals based on the presence or absence of the following traits^7^: females (divided by 1.5), current smoking (multiplied by 0.44), previous smoking (multiplied by 0.78), family history of PD (multiplied by 4.45), more than one cup of coffee per day (multiplied by 0.67), more than one alcoholic drink per week (multiplied by 0.9), constipation (multiplied by 2.34), anxiety or depression (multiplied by 1.86) and erectile dysfunction (multiplied by 3.8). The final odds for PD was converted to the probability scale using the equation:

$$Pr\left(PD\right)=\frac{Odds\left(PD\right)}{1+Odds\left(PD\right)}$$

To assess model performance as a covariate in logistic regression models, the PREDICT-PD estimate for liability was converted to the log odds scale.

#### Evaluation of model performance

To determine whether the PRS explained PD risk in UKB and whether it improved existing risk prediction models, we first divided the subset of European, unrelated UKB participants into a training set (30%) and a testing set (70%). The training set was used to select the PRS which explained the maximal PD risk.

To evaluate PRS performance in the training set comprising 451 prevalent PD cases and 100 446 controls, we used Nagelkerke’s pseudo-R^2^ metric comparing a full model (PD status ~Age+Sex+Townsend+PCs 1–4+PRS) to a null model (PD status ~Age+Sex+Townsend+PCs 1–4+PRS). Ninety-five per cent CIs were derived from 1000 bootstrap replicates using normal approximations as test statistics were approximately normally distributed. We selected the PRS with the highest absolute Nagelkerke’s pseudo-R^2^ for further validation.

To evaluate predictive model performance in the testing set, we calculated discrimination statistics (area under the curve (AUC)), calibration statistics and Nagelkerke’s pseudo-R^2^ using a variety of models (online supplementary figures).

10.1136/jnnp-2020-323646.supp2 Supplementary data

#### Gene–environment interactions

All analyses examining gene–environment interactions were conducted in the testing set to mitigate against bias from overfitting of the PRS. Interactions were assessed on both the additive and the multiplicative scales. Interaction on the additive scale was assessed by calculating the attributable proportion (AP) due to interaction. Additive interaction analyses were based on multivariable logistic regression models incorporating age at recruitment, sex, deprivation and the first four genetic principal components as confounders.^22^

For a logistic regression model of the form:

$$log\left(\frac{p}{1-p}\right)=\beta_{0}+\beta_{RF1}x+\beta_{RF2}y+\beta_{RF1*RF2}x\times y$$

in which (p/(1-p)) is the log odds of PD and are the values of exposure variables (e.g. childhood body size, smoking, PRS) and is the interaction term, then the Relative Excess Risk due to Interaction (RERI) can be calculated as:

$$RERI=exp\left(\beta_{RF1}+\beta_{RF2}+\beta_{RF1*RF2}\right)-exp\left(\beta_{RF1}\right)-exp\left(\beta_{RF2}\right)+1$$

The AP can be conceived of as the proportion of the disease in the doubly exposed group attributable to the interaction between the risk factors, that is,

$$AP=\frac{RERI}{exp\left(\beta_{RF1}+\beta_{RF2}+\beta_{RF1*RF2}\right)}$$

This model can be expanded to include confounding covariates, in which case the beta coefficients are adjusted for confounders. Derivation and further discussion of the advantages of this method over Rothman’s initial description can be found in Knol *et al*.^22^ We restricted this analysis to participants with genetically European ancestry determined by both self-report (‘Caucasian’ in UKB data) and genetic ethnic grouping. For interaction analyses using the PRS, the covariates were age, sex, current deprivation and the first four genetic principal components. The PRS was transformed using the inverse normal transformation and treated as a continuous variable for these analyses. For the menarche analysis, age at menarche was also transformed using the inverse normal transformation. CIs for the AP were estimated using bootstrap resampling of the entire dataset with replacement for 5000 iterations.^22^ Ninety-five per cent CIs were derived from the 2.5th and 97.5th percentile values. Interactions on the multiplicative scale were assessed using a logistic regression model incorporating an interaction term. The presence of multiplicative interaction was assessed using the likelihood ratio test, with an overall FDR threshold at 5%.

### Computing

This research was supported by the High-Performance Cluster computing network hosted by Queen Mary University of London.^23^

Statistical analyses were performed in R V.3.6.1. Extraction of European individuals from the 1000 genomes reference genome was conducted using vcftools. Construction of the PRS, application of the PRS to individuals and quality control were performed in PLINK V.1.9 and PLINK V.2.00. Code used for this paper is available at https://github.com/benjacobs123456/PD_UKB_PRS.

## Results

### Demographics of cases and controls

Phenotype data were available for 2127 individuals with PD, of whom 1276 were diagnosed after enrolment (incident cases) and 500 406 controls. After the exclusions outlined for the genetic analyses, 1342 of the 2127 individuals with PD remained and 801 of the 1276 incident PD cases remained. Of the 1276 incident cases, 1243 (97.4%) had a Hospital Episode Statistics coded diagnosis of PD and 33 (2.59%) individuals had a self-reported diagnosis only. Demographic characteristics of individuals with PD (both prevalent and incident cases) and controls are shown in online supplementary table 2. In unadjusted comparisons, cases with PD were more likely to be older (mean age at recruitment 62.7 years, SD 5.49), male (61.6% male), born in the UK, of white ethnicity, and less deprived. Age at PD diagnosis was consistent with published estimates (median 66.1 years, IQR 59.5–71.7).^24^ Median follow-up time was similar for cases (median 12.01 years, IQR 11.01–13.01) and controls (median 12.01 years, IQR 11.01–13.00).

### Risk factors and prodromal features

**Figure 1:**
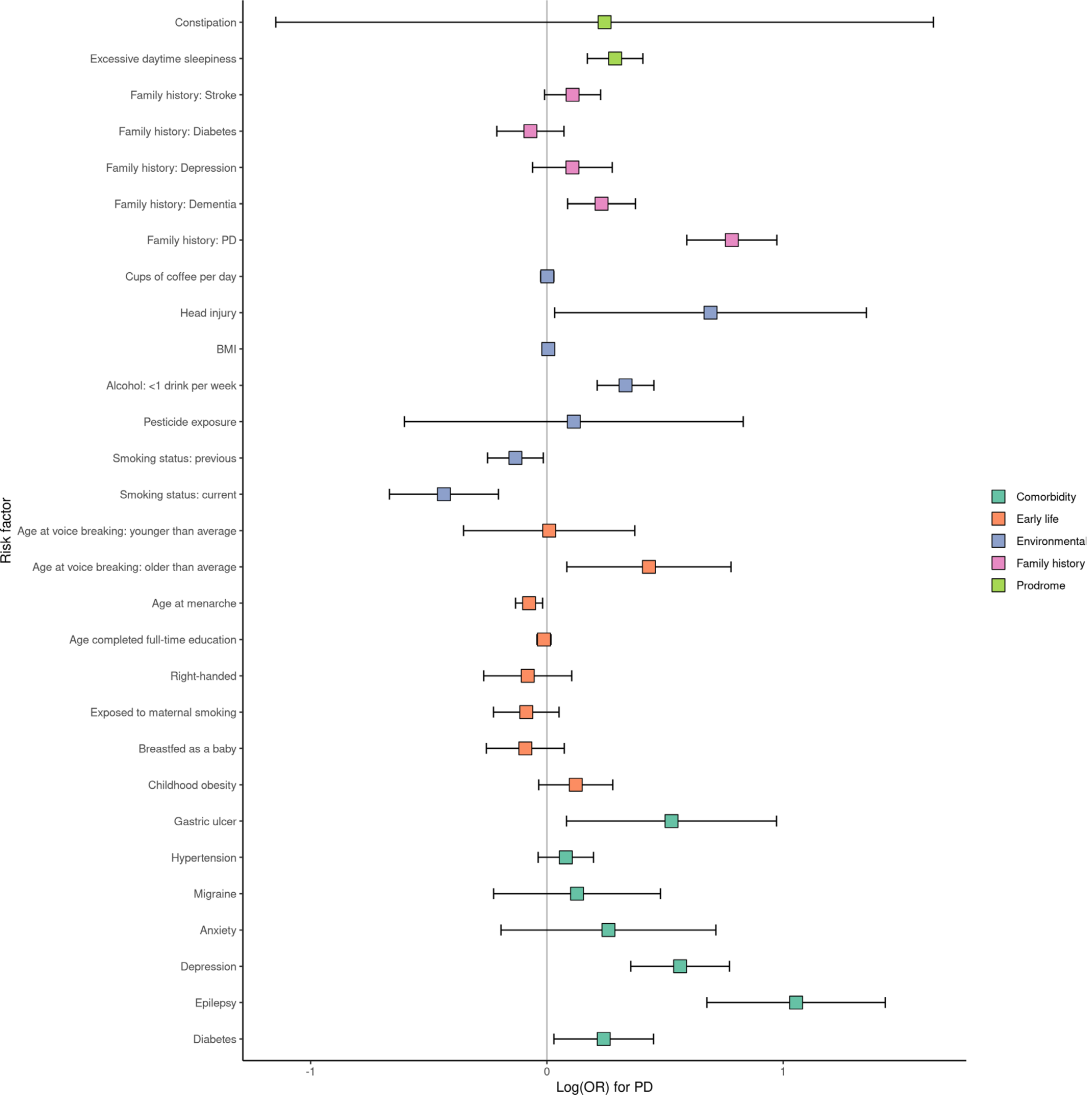
Associations of risk factors and incident cases of PD. Point estimates for association are depicted as log ORs and 95% CIs. Estimates of association were derived from logistic regression models adjusting for age, sex, Townsend deprivation index at recruitment and ethnicity. BMI, body mass index; PD, Parkinson’s disease.

There was strong evidence of a positive association (FDR<0.05) between incident PD diagnosis and having a family history of PD, not smoking, low alcohol consumption (<1 drink/week), depression, excessive daytime sleepiness, a family history of dementia, epilepsy and earlier menarche. There was weaker evidence (FDR<0.10) for an association between PD and having had peptic ulcer disease or diabetes mellitus (figure 1, table 1). Effect estimates and precision did not alter substantially in a multivariable model including all strongly associated (FDR<0.05) risk factors (age of menarche was excluded to allow inclusion of both sexes; table 2). Exclusion of non-white individuals, individuals <60 years old at recruitment, self-reported PD cases and use of a matched 4:1 case‒control design did not alter these results (online supplemental figures 1‒4).

**Table 1:** Associations of risk factors with incident PD status

Risk factor	Category	OR	Lower 95% CI	Upper 95% CI	P value	Likelihood ratio FDR Q
Family history: PD	Family history	2.19	1.81	2.65	3.62E-13	9.78E-12
Alcohol: <1 drink per week	Environmental	1.39	1.24	1.57	8.96E-08	1.21E-06
Depression	Comorbidity	1.76	1.43	2.17	9.40E-07	8.46E-06
Epilepsy	Comorbidity	2.87	1.97	4.19	2.58E-06	1.39E-05
Excessive daytime sleepiness	Prodrome	1.33	1.19	1.50	2.07E-06	1.39E-05
Smoking status: current	Environmental	0.65	0.51	0.81	2.34E-04	0.001
Smoking status: previous	Environmental	0.88	0.78	0.98	2.34E-04	0.001
Family history: dementia	Family history	1.26	1.09	1.45	0.002	0.008
Age at menarche	Early life	0.93	0.88	0.98	0.010	0.032
Diabetes	Comorbidity	1.27	1.03	1.57	0.030	0.086
Gastric ulcer	Comorbidity	1.69	1.09	2.64	0.032	0.086
Age at voice breaking: older than average	Early life	1.01	0.70	1.45	0.073	0.156
Age at voice breaking: younger than average	Early life	1.54	1.09	2.18	0.073	0.156
Family history: stroke	Family history	1.11	0.99	1.26	0.075	0.156
Head injury	Comorbidity	2.00	1.03	3.87	0.064	0.156
Childhood obesity	Early life	1.13	0.97	1.32	0.132	0.255
Hypertension	Comorbidity	1.08	0.96	1.22	0.182	0.328
Exposed to maternal smoking	Early life	0.92	0.80	1.05	0.213	0.343
Family history: depression	Family history	1.11	0.94	1.32	0.216	0.343
Anxiety	Comorbidity	1.30	0.82	2.04	0.281	0.400
Breastfed as a baby	Early life	0.91	0.77	1.08	0.281	0.400
Family history:diabetes	Family history	0.93	0.81	1.07	0.331	0.447
BMI	Environmental	1.01	0.99	1.02	0.363	0.467
Age completed full time education	Early life	0.99	0.96	1.02	0.403	0.473
Right-handed	Early life	0.92	0.77	1.11	0.398	0.473
Migraine	Comorbidity	1.14	0.80	1.62	0.488	0.549
Constipation	Prodrome	1.28	0.32	5.13	0.741	0.789
Pesticide exposure	Environmental	1.12	0.55	2.30	0.759	0.789
Cups of coffee per day	Environmental	1.00	0.97	1.03	0.918	0.918

Output is from logistic regression models of the form PD status ~Age+Sex+Deprivation+Ethnicity+risk factor. P values are asymptotic p values calculating from the z statistic. Likelihood ratio test Q values represent the local FDR-corrected p value for the likelihood ratio test comparing model fit with and without the risk factor term (ie, compared with a null model consisting only of the covariates age, sex, ethnicity and deprivation).

BMI, body mass index; FDR, false discovery rate; PD, Parkinson’s disease.

**Table 2:** Associations of risk factors with incident PD status in a combined model adjusting for all other risk factors

Risk factor	OR	Lower 95% CI	Upper 95% CI	P value
Family history: PD	2.13	1.76	2.58	1.38E-14
Depression	1.72	1.40	2.12	3.93E-07
Epilepsy	2.65	1.80	3.89	7.35E-07
Alcohol: <1 drink/week	1.34	1.19	1.51	2.53E-06
Excessive daytime sleepiness	1.29	1.14	1.45	3.01E-05
Current smoking	0.65	0.52	0.82	3.10E-04
Family history: dementia	1.22	1.05	1.40	0.008
Age at menarche	0.94	0.88	0.99	0.026
Previous history of smoking	0.90	0.80	1.01	0.070

Output is from logistic regression models of the form PD status~Age+Sex+Deprivation+Ethnicity+risk factors. P values are asymptotic p values calculating from the z statistic. In this model, all covariates found to be associated with PD at FDR<0.05 were included. The estimate for menarche is derived from a model without a sex term as it was restricted to females.

FDR, false discovery rate; PD, Parkinson’s disease.

### Validation of PREDICT-PD risk algorithm

**Figure 2:**
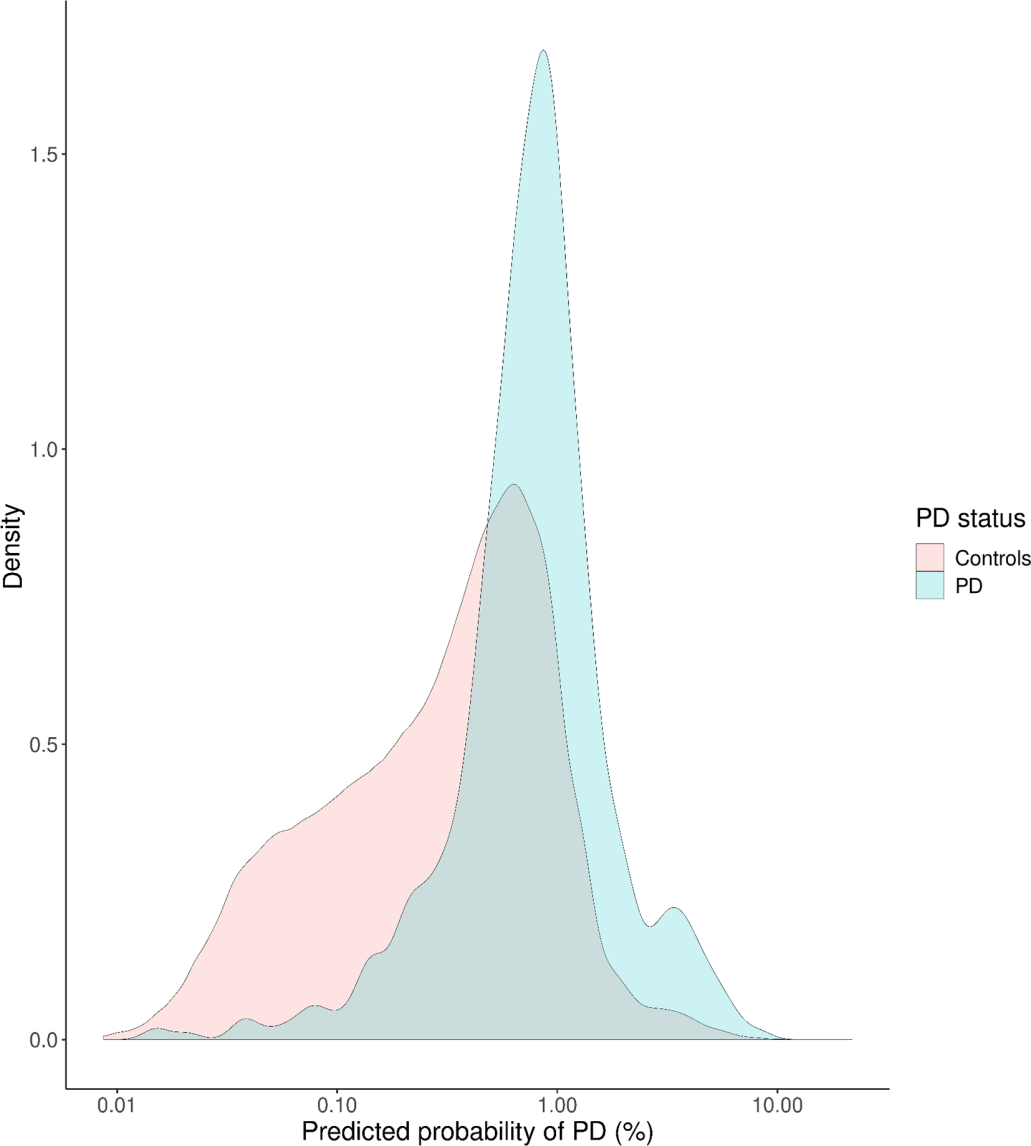
PREDICT-PD determined probability (on the absolute risk scale) of PD, determined at recruitment, for individuals who would go on to develop PD (incident cases) and those who would not (controls). PD, Parkinson’s disease.

In the whole cohort, the PREDICT-PD algorithm had some discriminative ability for distinguishing incident PD cases from controls (Nagelkerke’s pseudo-R^2^ 0.060, likelihood ratio p<2×10^−16^, online supplementary figures 5 and 6). The median predicted probability of PD was 2.31× higher among incident PD cases than controls (median risk 0.81%, IQR 0.61 in cases vs median risk 0.35%, IQR 0.57 in controls; figure 2).

### Genetic risk score

**Figure 3:**
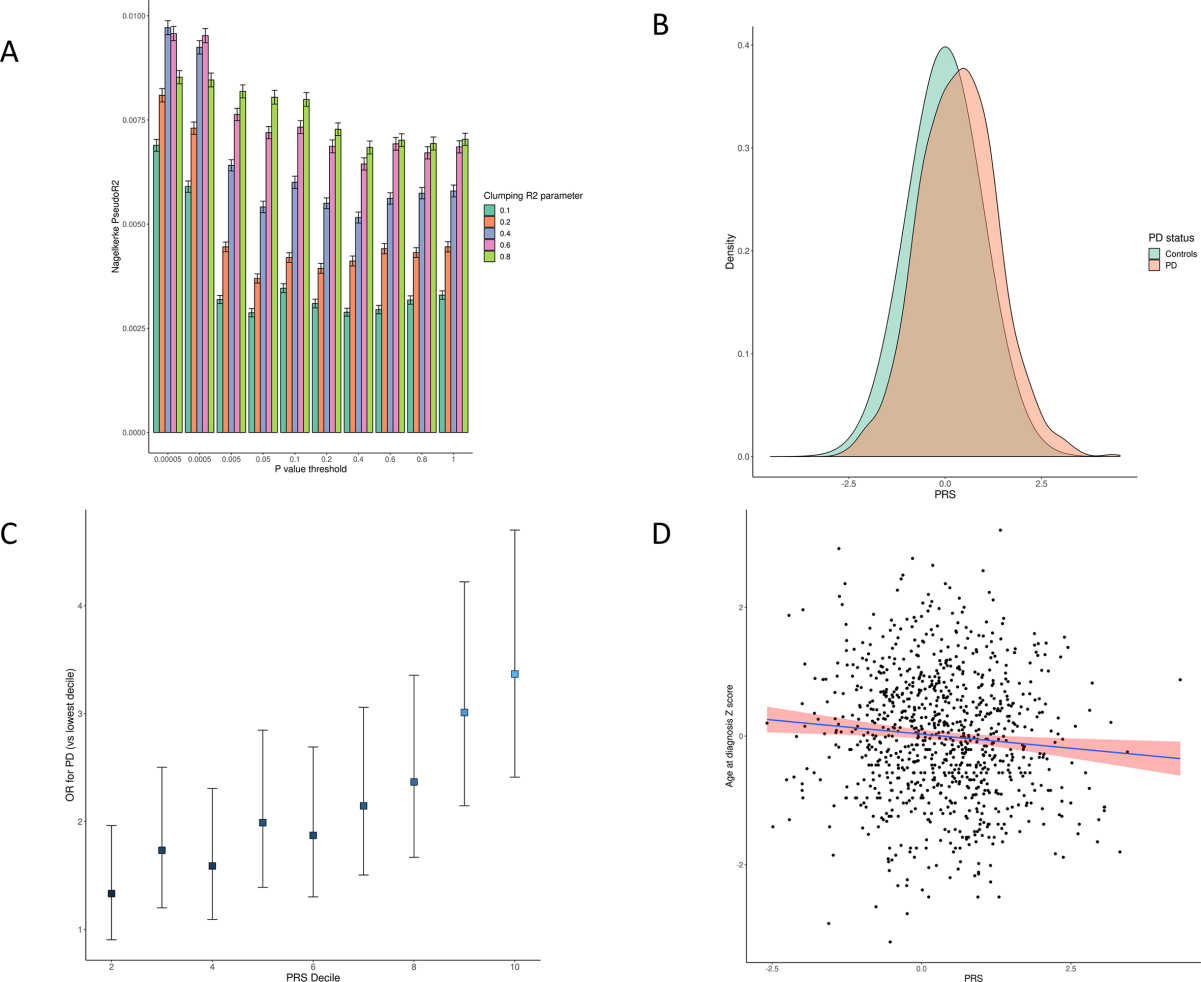
(A) Several candidate Polygenic Risk Scores (PRSs) were created using summary statistics from the Meta5 PD GWAS excluding UKB participants. For each candidate PRS, the degree of variation in PD risk explained was estimated using Nagelkerke’s pseudo-R2 metric. 95% CIs were derived from 1000 bootstrap resamples of the training dataset. As test statistics were approximately normally distributed, 95% CIs were derived from the normal distribution (mean±1.96 x SE). (B) Normalised PRS values for incident PD cases and controls. (C) OR of PD by PRS decile compared with lowest PRS decile. (D) Correlation between increasing PRS and earlier age at PD diagnosis. GWAS, genome-wide association studies; PD, Parkinson’s disease.

After ancestry and relatedness exclusions (online supplementary figures 7 and 8), we divided the cohort into training (30%) and testing (70%) sets to mitigate against overfitting. We evaluated the performance of various polygenic scores in the training set, which consisted of 451 PD cases altogether (including prevalent cases) and 100 446 controls. Within the training set, the best-fitting PRS had the following parameters: p value threshold <5×10^−5^, clumping R^2^ threshold 0.4, Nagelkerke’s pseudo-R^2^ 0.0097, 95% CI 0.0095 to 0.0099, 983 SNPs included (figure 3, online supplementary tables 4 and 5). We then evaluated the performance of this PRS in the testing set, which consisted of 600 incident PD cases, 1007 PD cases altogether (including prevalent cases) and 234 418 controls. The selected PRS had predictive power for incident PD in the testing set comparable with that in the training set (Nagelkerke’s pseudo-R^2^ 0.0099, likelihood ratio test p<2×10^−16^; figure 3, supplementary figures 5 and 6).

Individuals in the highest PRS decile had approximately 3.4× increased risk of PD (OR 3.37, 95% CI 2.41 to 4.70) compared with the lowest decile (figure 3, online supplementary table 7 for case and control counts in each decile). Higher PRS scores were also associated with age at PD diagnosis in a linear model adjusting for age, sex and the first four genetic principal components (PCs; beta −0.060 per 1-SD increase in PRS, p=0.016, figure 3). This estimate is similar to a published estimate from the IPDGC.^25^

Inclusion of the PRS improved model fit for PD risk compared with a null model including only the PREDICT-PD algorithm (which incorporates age and sex), and the first four genetic PCs (Nagelkerke pseudo-R^2^ 0.005, p=2.11×10^−9^, online supplementary figures 5 and 6). We modified this PRS to exclude all variants within known PD genomic risk loci (see Nalls *et al* 2019 and online supplementary table 6): for each risk locus, all variants 1 MB either side of the lead SNP were removed. This modified PRS did not explain additional PD liability compared with the PREDICT-PD algorithm alone (Nagelkerke pseudo-R^2^ 2.94×10^–6^, p=0.89), suggesting that variation within established PD risk loci accounts for the majority of the predictive power of the PRS.

### Interactions

**Figure 4:**
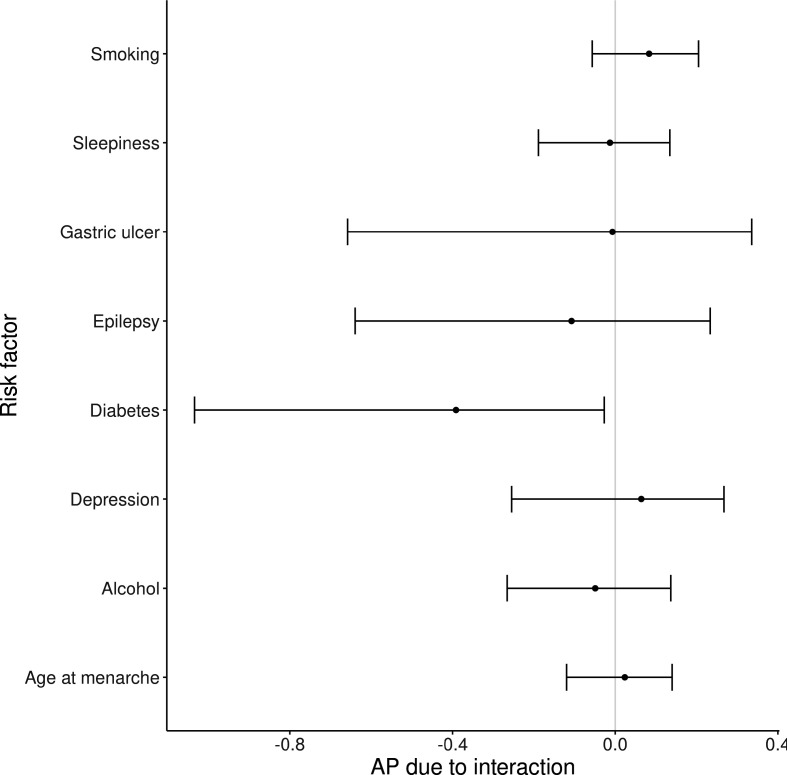
Interactions between risk factors for PD and the PD PRS were estimated using the attributable proportion due to interaction. Point estimates for the AP and 95% CIs are shown. PD, Parkinson’s disease; PRS, Polygenic Risk Score.

Interactions between the PRS and eight risk factors/prodromal symptoms found to be associated with PD risk at FDR<0.10 were analysed (family history of PD and dementia were not included because of the potential overlap with the PRS, and smoking exposure included as never/ever smoker). Although there was no evidence for multiplicative interactions surpassing the FDR of 5%, there was nominal evidence (p<0.05) for a negative multiplicative interaction between diabetes and the PRS (beta −0.40, p=0.026, online supplementary table 7). We also found evidence of interaction on the additive scale between the PRS and diabetes (AP −0.39, 95% CI −1.03 to−0.03; figure 4). These results suggest that diabetes is a more potent risk factor among people at low genetic risk of PD.

## Discussion

In this study, we used data from UKB to determine risk factors and protective factors for PD, demonstrate the predictive power of a PRS relative to a previously validated risk prediction algorithm and provide novel evidence for differential effects of diabetes on PD risk depending on prior genetic risk.

We observed strong associations with incident PD for several well-established risk and protective factors. In a model adjusted for age, sex, ethnicity and current deprivation, risk factors for incident PD were having a family history of PD, not smoking, low alcohol consumption (<1 drink/week), depression, excessive daytime sleepiness, a family history of dementia, epilepsy and earlier age of menarche. All of these factors remained associated with incident PD when modelled jointly. We also found suggestive evidence of association (FDR<0.10) for gastric ulceration and diabetes mellitus. There was no evidence in this cohort that anxiety, BMI, constipation, pesticide exposure or coffee consumption influence PD risk.

In a recent paper from some members of this group, novel cross-sectional associations with PD were reported for migraine and epilepsy.^5^ Here, we have not only replicated the association with epilepsy but also demonstrated a temporal relationship with incident PD. Whether the association is driven by epilepsy, chronic use of antiepileptic drugs or residual confounding remains to be determined. There was no convincing association with migraine in the present study.

The association of earlier age at menarche with PD is novel and intriguing. In addition, we observed weak evidence for a similar effect of earlier age at voice breaking in males, suggesting that earlier pubertal timing in both sexes may increase PD risk. The sex dimorphism in PD incidence suggests possible protective roles for female sex hormones or possible harmful roles for male sex hormones.^26^ In animal models of PD, sex hormones have pleiotropic effects, which are inconsistent between studies.^26–30^ The broad consensus from animal models and epidemiological studies of menopausal timing and PD^31 32^ is that oestrogens may be neuroprotective. In this context, our findings are counterintuitive, as earlier menarche should predispose towards greater lifetime oestrogen exposure. It is possible that the observational association between earlier puberty and PD risk is driven by residual confounding. Both the genetic and the environmental determinants of pubertal timing may confound the relationship with PD risk.^33^ Thus, we would interpret this association with caution and encourage replication in other settings.

Next, we demonstrated that a basic risk algorithm previously developed in the PREDICT-PD study could be used to predict incident cases of PD in UKB with moderate discriminative capability (AUC 0.76).^34^ Whether risk prediction algorithms based on clinical parameters can be enhanced by use of genetic risk scores is an area of considerable scientific and clinical interest.^35^ To answer this question for PD in UKB, we first created several candidate PRS using association statistics from the largest PD GWAS to date. Next, we selected the best fitting PRS using a subset of the UKB data (30% training set). We then validated the predictive performance of this PRS in the remaining 70% of the cohort (testing set). We show that inclusion of a PRS improves predictive performance over the PREDICT-PD algorithm in UKB; however, the absolute incremental advantage is very small, consistent with previous similar efforts in other disease areas.^36–38^ Although this small increment is not helpful for clinical risk prediction, this approach can be used to enrich cohort study populations for individuals at higher risk of developing PD.^39^ The use of a PRS with and without other risk factors for PD has been previously validated in a large case–control setting,^40^ but there are limited examples of application in a population setting such as we have done.^41^

Finally, we undertook some preliminary study of the role of gene–environment interactions for PD in UKB. We compared how the association of the various exposures in the model varied across strata of genetic risk. Prior to this, simple gene–environment interaction studies have been undertaken to investigate the effect modification by a single gene or locus on an environmental risk factor.^42 43^ Here, we used the PRS to show that the association with diabetes is potentially modified such that it plays a bigger role as a risk factor in those at lower genetic risk and may have (or its treatment may have) a protective effect in those at higher genetic risk.

This observation is especially interesting in the context of recent phase II clinical trial data showing that the antihyperglycaemic drug exenatide (a glucagon-like peptide-1 agonist) had efficacy in reducing off-medication motor symptoms in PD.^44^ Potentially shared cellular signalling pathways for this group of drugs and PD pathophysiology have also been highlighted.^45^ It is conceivable that our results may therefore reflect confounding by drug treatment—that is, if the treatment of diabetes differs systematically between individuals at high and low risk of PD. As genetic risk for PD (quantified by genome-wide PRS) may itself be a surrogate for subtle ethnic variation, socioeconomic status and other confounders, so it is plausible that there could be real differences in access to particular antidiabetes medications between strata of the PRS. If the effect of antidiabetic drugs on PD is modified dramatically by prior genetic risk for PD, it may be possible to select individuals who are more likely to benefit from these drugs in phase III trials. Validation of our results and exploration of the mechanism for this interaction are required before translation into trial selection criteria.

We have previously observed markedly different effects of diabetes on PD risk depending on study design.^4^ While survival bias may account for some of the observed variability in effect estimates comparing case–control and cohort studies, genetic population stratification may also be an important source of variation as indicated here. Correcting for genetic principal components should mitigate confounding due to population stratification but may not eliminate it. The importance of genetic stratification for PD intervention studies has been recently explored,^38^ and in the current study, we demonstrate further evidence for why genetic stratification is an important consideration.

The strengths of this study are that we used a very large sample size to measure risk and protective factors for PD, as well as to externally validate the PREDICT-PD algorithm in a cohort where incident cases are accruing. The prospective design reduces the likelihood of reverse causation but in diseases with a long prodromal phase (such as PD), reverse causation cannot be completely dispelled. However, for the purpose of predicting incident cases, whether factors in the model are true exposures or prodromal features is less concerning. As many of the exposures vary by age and gender, we adjusted all exposure variables for important confounding factors. We have previously surveyed a subgroup of the PREDICT-PD participants and found that less than 5% were participants in UKB, hence overlap in populations is minimal. The latest PD GWAS used data from PD cases in UKB and the controls, but we used summary statistics which excluded UKB cases, UKB controls and UKB proxy cases to avoid sample overlap and overfitting models.

General limitations are that the definition of incident PD cases in this setting relied to a small extent on self-report and several important risk factors for incident PD were inadequately captured. Both of these factors may lead to bias and imprecision in the effect estimates. Another important consideration is the generalisability of UKB. Recruitment into the UKB cohort was voluntary with 5.5% of those invited ultimately joining. Comparing the UKB population to UK Census and representative cross-sectional survey data shows that typically UKB participants were more likely to be White British (by self-report), female, older and from more affluent areas. Within the cohort rates of smoking, obesity and daily drinking were less than that in the general UK population.^46^ A major concern is that such non-random recruitment may introduce spurious associations and destroy true associations due to collider bias.^47^ We excluded participants of non-European ancestry from analyses including the PRS, so these results are likely to have limited applicability to other populations. Although overfitting of the PRS is still a possibility, we mitigate this by strictly dividing the cohort into training and testing sets for tuning and testing the PRS, respectively.

To conclude, we have confirmed several well-established risk and protective factors for PD and shed further light on several novel associations (migraine, epilepsy, earlier menarche). We have externally validated the basic PREDICT-PD algorithm and extended this approach to incorporate population-level common genetic variation. We have modelled interactions between environmental factors, comorbidities and polygenic risk to demonstrate how interplay between genetic and other risk factors may contribute to PD risk. These findings could have implications for risk stratification of individuals for studies examining the ‘pre-diagnostic’ phase of PD and for our understanding of how genetic variation and other risk factors interact in the pathogenesis of PD.
