# Supplementary material for: Parkinson’s disease determinants, prediction and gene–environment interactions in the UK Biobank
Source: J Neurol Neurosurg Psychiatry. 2020 Sep 14;91(10):1046–54. doi: 10.1136/jnnp-2020-323646 (PMC7509524; doi:10.1136/jnnp-2020-323646)
Supplement: Supplementary data [file jnnp-2020-323646supp001.pdf]

**Supplementary table 1: definition of exposure variables.**

| Exposure | UK Biobank Field ID | UK Biobank Description | ICD 10 code |
|---|---|---|---|
| **Age** | 21022 | Age at recruitment | N/a |
| **Sex** | 31 | Sex | N/a |
| **Education** | 845 | Age completed full time education | N/a |
| **Townsend deprivation index** | 189 | Townsend deprivation index at recruitment | N/a |
| **Ethnicity** | 21000 | Ethnic background | N/a |
| **Country of birth** | 1647 | Country of birth (UK/elsewhere) | N/a |
| **Parkinson's disease** | HES coding, Non-cancer illness code, self-reported | N/a | G20 |
| **Constipation** | HES coding, Non-cancer illness code, self-reported | N/a | K59.0 |
| **Depression** | HES coding, Non-cancer illness code, self-reported | N/a | F32.0, F32.1, F32.2, F32.3, F32.8, F32.9, F33.0, F33.1, F33.2, F33.3, F33.4, F33.8, F33.9 |
| **Anxiety** | HES coding, Non-cancer illness code, self-reported | N/a | F41.2, F41.3, F41.8, F41.9 |
| **Hypertension** | HES coding, Non-cancer illness code, self-reported | N/a | I10 |
| **Diabetes** | HES coding, Non-cancer illness code, self-reported | N/a | E10.0, E10.1, E10.2, E10.3, E10.4, E10.5, E10.6, E10.7, E10.8, E10.9, E11.0, E11.1, E11.2, E11.3, E11.4, E11.5, E11.6, E11.7, E11.8, E11.9, E12.0 |
| **Gastric ulcer** | HES coding, Non-cancer illness code, self-reported | N/a | K25.0, K25.1, K25.2, K25.3, K25.4, K25.5, K25.6, K25.7, K25.9 |
| **Migraine** | HES coding, Non-cancer illness code, self-reported | N/a | G43.0, G43.1 |

| | | | |
|---|---|---|---|
| **Shoulder stiffness** | HES coding, Non-cancer illness code, self-reported | N/a | M25.61 |
| **Shoulder pain** | HES coding, Non-cancer illness code, self-reported | N/a | M25.51 |
| **Hyposmia** | HES coding, Non-cancer illness code, self-reported | N/a | R43.0 |
| **Erectile dysfunction** | HES coding, Non-cancer illness code, self-reported | N/a | N48.4 |
| **Head injury** | HES coding, Non-cancer illness code, self-reported | N/a | S02.0, S02.1, S02.2, S02.3, S02.4, S02.6, S02.7, S02.8, S02.9, S06.0, S06.1, S06.2, S06.3, S06.4, S06.5, S06.6, S06.8, S06.9 |
| **Family history: Parkinson's disease** | 20107, 20110, 20111 | Illnesses of father, Illnesses of mother, Illnesses of siblings | N/a |
| **Family history: Depression** | 20107, 20110, 20111 | Illnesses of father, Illnesses of mother, Illnesses of siblings | N/a |
| **Family History: Dementia** | 20107, 20110, 20111 | Illnesses of father, Illnesses of mother, Illnesses of siblings | N/a |
| **Family History: Stroke** | 20107, 20110, 20111 | Illnesses of father, Illnesses of mother, Illnesses of siblings | N/a |
| **Family History: Diabetes** | 20107, 20110, 20111 | Illnesses of father, Illnesses of mother, Illnesses of siblings | N/a |
| **Smoking status** | 20116 | Smoking status | N/a |
| **Cups of coffee per day** | 1498 | Coffee intake | N/a |
| **Alcohol** | 1558 | Alcohol intake frequency | N/a |
| **Body mass index** | 21001 | Body mass index | N/a |
| **Daytime sleepiness** | 1220 | Daytime dozing / sleeping (narcolepsy) | N/a |
| **Pesticides** | 22614 | Worked with pesticides | N/a |
| **Breast fed** | 1677 | Breast fed as a baby | N/a |

| | | | |
|---|---|---|---|
| **Childhood obesity** | 1687 | Comparative body size at age 10 | N/a |
| **Handedness** | 1707 | Handedness (chirality/laterality) | N/a |
| **Maternal smoking** | 1787 | Maternal smoking around birth | N/a |
| **Age of menarche** | 2714 | Age when periods started (menarche) | N/a |
| **Age of voice breaking** | 2385 | Relative age voice broke | N/a |

**Supplementary table 2: demographics of PD cases and controls. Continuous variables are depicted as mean (SD). Categorical variables are depicted as n (%). Univariate comparisons reflect chi-squared tests for categorical variables and unpaired two-sample t tests for continuous variables.**

| *Prevalent cases* | | | |
|---|---|---|---|
| **Trait** | **Control** | **Cases** | **P value** |
| **Age** | 56.5 (8.09) | 62.74 (5.49) | <2E-16 |
| **Age completed full-time education** | 16.74 (4.81) | 16.43 (2.44) | 1.75E-06 |
| **Townsend deprivation index** | -1.29 (3.1) | -1.45 (3.07) | 0.019181457 |
| **Sex** | | | 1.10E-49 |
| Female | 272578 ( 54.47 %) | 817 ( 38.41 %) | |
| Male | 227819 ( 45.53 %) | 1310 ( 61.59 %) | |
| **Ethnicity** | | | 6.84E-05 |
| White | 470670 ( 94.6 %) | 2039 ( 96.59 %) | |
| Non-white | 26841 ( 5.4 %) | 72 ( 3.41 %) | |
| **Country of birth** | | | 2.94E-04 |
| UK | 458838 ( 92.02 %) | 1991 ( 94.18 %) | |
| Non-UK | 39788 ( 7.98 %) | 123 ( 5.82 %) | |
| | | | |
| *Incident cases* | | | |
| **Trait** | **Control** | **Cases** | **P value** |
| **Age** | 56.5 (8.09) | 63.09 (5.47) | 5.95E-251 |
| **Age completed full-time education** | 16.74 (4.81) | 16.36 (2.31) | 1.41E-06 |
| **Townsend deprivation index** | -1.29 (3.1) | -1.28 (3.14) | 0.84498473 |
| **Sex** | | | 4.30E-31 |
| Female | 272578 ( 54.47 %) | 488 ( 38.24 %) | |
| Male | 227819 ( 45.53 %) | 788 ( 61.76 %) | |
| **Ethnicity** | | | 0.003093495 |
| White | 470670 ( 94.6 %) | 1222 ( 96.52 %) | |
| Non-white | 26841 ( 5.4 %) | 44 ( 3.48 %) | |
| **Country of birth** | | | 0.003124537 |
| UK | 458838 ( 92.02 %) | 1194 ( 94.31 %) | |
| Non-UK | 39788 ( 7.98 %) | 72 ( 5.69 %) | |

**Supplementary table 3: SNPs included in the 'best' PRS, as determined by highest pseudo-$R^2$ in the training set. Annotations are given in hg38 and nearest genes are derived from Ensembl Variant Event Predictor.**

| RSID | CHR:BP (hg38) | Nearest gene |
|---|---|---|
| rs845250 | 1:7423291-7423291 | CAMTA1 |
| rs77901899 | 1:62271453-62271453 | KANK4 |
| rs148303184 | 1:62567432-62567432 | DOCK7 |
| rs4655643 | 1:66624416-66624416 | SGIP1 |
| rs11208947 | 1:66689781-66689781 | SGIP1 |
| rs278839 | 1:77642189-77642189 | AC118549.1 |
| rs11164880 | 1:93192539-93192539 | CCDC18 |
| rs114809584 | 1:96889283-96889283 | - |
| rs9728526 | 1:145718303-145718303 | CD160 |
| rs74916032 | 1:147535311-147535311 | - |
| rs201694776 | 1:152616206-152616206 | LCE3B |
| rs12738393 | 1:154245167-154245167 | UBAP2L |
| rs34450945 | 1:154316872-154316872 | AQP10 |
| rs114853077 | 1:154880259-154880259 | - |
| rs11264304 | 1:155060841-155060841 | ADAM15 |
| rs12743272 | 1:155060858-155060858 | ADAM15 |
| rs35749011 | 1:155162560-155162560 | - |
| rs12748814 | 1:155340652-155340652 | ASH1L |
| rs71628684 | 1:155715686-155715686 | DAP3 |
| rs35603727 | 1:156038197-156038197 | UBQLN4 |
| rs10737170 | 1:156094089-156094089 | LMNA |
| rs2853643 | 1:156215040-156215040 | PMF1-BGLAP |
| rs12748659 | 1:156330207-156330207 | CCT3 |
| rs4326638 | 1:161419170-161419170 | AL592295.4 |
| rs61802091 | 1:161419856-161419856 | AL592295.4 |
| rs114797774 | 1:161498640-161498640 | - |
| rs4657041 | 1:161509069-161509069 | FCGR2A |
| rs11810143 | 1:161510859-161510859 | FCGR2A |
| rs79277331 | 1:161537099-161537099 | FCGR3A |
| rs144055958 | 1:161563618-161563618 | AL590385.2 |
| rs17411858 | 1:161647726-161647726 | - |
| rs235487 | 1:171562020-171562020 | PRRC2C |
| rs76357776 | 1:171674923-171674923 | PFN1P1 |
| rs10913559 | 1:171732347-171732347 | VAMP4 |
| rs10913622 | 1:171760535-171760535 | Z98751.2 |
| rs57476157 | 1:171803313-171803313 | AL031864.1 |
| rs12141455 | 1:193352957-193352957 | LINC01031 |
| rs55922528 | 1:194585600-194585600 | - |
| rs78203442 | 1:194766300-194766300 | - |
| rs116592662 | 1:194863221-194863221 | AL353072.2 |
| rs4915470 | 1:200965282-200965282 | KIF21B |
| rs296562 | 1:200976146-200976146 | KIF21B |

| | | |
|---|---|---|
| rs61819408 | 1:204794161-204794161 | - |
| rs12078075 | 1:205194670-205194670 | DSTYK |
| rs141969842 | 1:205674792-205674792 | SLC45A3 |
| rs4245718 | 1:205683344-205683344 | SLC45A3 |
| rs7545345 | 1:205721813-205721813 | NUCKS1 |
| rs11240565 | 1:205753830-205753830 | NUCKS1 |
| rs141145750 | 1:205756888-205756888 | - |
| rs73080374 | 1:205783562-205783562 | AC119673.2 |
| rs12740609 | 1:205787407-205787407 | SLC41A1 |
| rs114984342 | 1:205839745-205839745 | PM20D1 |
| rs77133845 | 1:226694124-226694124 | ITPKB |
| rs4653767 | 1:226728377-226728377 | ITPKB |
| rs149905768 | 1:226786527-226786527 | RPS27P5 |
| rs61835654 | 1:226795629-226795629 | - |
| rs12075077 | 1:226911075-226911075 | COQ8A |
| rs55925257 | 1:227871638-227871638 | - |
| rs147393995 | 1:228039106-228039106 | WNT3A |
| rs34249947 | 1:228136466-228136466 | GUK1 |
| rs7523171 | 1:232460383-232460383 | SIPA1L2 |
| rs6672473 | 1:232507091-232507091 | SIPA1L2 |
| rs10797576 | 1:232528865-232528865 | SIPA1L2 |
| rs943140 | 1:232539286-232539286 | SIPA1L2 |
| rs9428576 | 1:243498725-243498725 | AKT3 |
| rs12408455 | 1:243578639-243578639 | AKT3 |
| rs17369299 | 10:15479880-15479880 | - |
| rs76761646 | 10:15485089-15485089 | - |
| rs7077871 | 10:15496004-15496004 | - |
| rs10906914 | 10:15507613-15507613 | - |
| rs79305906 | 10:22679230-22679230 | PIP4K2A |
| rs2754493 | 10:38362521-38362521 | HSD17B7P2 |
| rs2489678 | 10:42458987-42458987 | CCNYL2 |
| rs11239219 | 10:44720664-44720664 | - |
| rs10857613 | 10:48621632-48621632 | ARHGAP22 |
| rs17721694 | 10:50407533-50407533 | SGMS1 |
| rs72809473 | 10:75695702-75695702 | LRMDA |
| rs846578 | 10:76739750-76739750 | - |
| rs111691636 | 10:77615783-77615783 | KCNMA1 |
| rs3781273 | 10:93643216-93643216 | PDE6C |
| rs142328600 | 10:94008085-94008085 | - |
| rs10882978 | 10:97663991-97663991 | PI4K2A |
| rs3740484 | 10:100987606-100987606 | SEMA4G |
| rs118165209 | 10:101999888-101999888 | ARMH3 |
| rs67497633 | 10:102055738-102055738 | ARMH3 |
| rs117507580 | 10:102182118-102182118 | - |
| rs10748818 | 10:102255522-102255522 | GBF1 |
| rs79931565 | 10:102415892-102415892 | PSD |

| | | |
|---|---|---|
| rs117542248 | 10:117751683-117751683 | LINC02674 |
| rs142479119 | 10:119419271-119419271 | GRK5 |
| rs4752331 | 10:119564464-119564464 | AC012468.1 |
| rs11592336 | 10:119611822-119611822 | - |
| rs188789342 | 10:119612816-119612816 | - |
| rs2577339 | 10:119662970-119662970 | BAG3 |
| rs196325 | 10:119669316-119669316 | BAG3 |
| rs3858339 | 10:119676556-119676556 | BAG3 |
| rs72842211 | 10:119678699-119678699 | BAG3 |
| rs932269 | 10:119680164-119680164 | BAG3 |
| rs113770268 | 10:119695890-119695890 | - |
| rs4751750 | 10:119720871-119720871 | - |
| rs117896735 | 10:119776815-119776815 | INPP5F |
| rs61870572 | 10:119887396-119887396 | MCMBP |
| rs12241478 | 10:119907508-119907508 | SEC23IP |
| rs1052289 | 10:119940743-119940743 | SEC23IP |
| rs117997675 | 10:119995603-119995603 | NACAP2 |
| rs72828546 | 10:120083083-120083083 | - |
| rs3117448 | 10:132351935-132351935 | LRRC27 |
| rs117011293 | 11:527311-527311 | HRAS |
| rs4963136 | 11:549119-549119 | LRRC56 |
| rs10835060 | 11:1466913-1466913 | BRSK2 |
| rs10840502 | 11:2179719-2179719 | - |
| rs7933395 | 11:7508194-7508194 | OLFML1 |
| rs112980556 | 11:10518378-10518378 | RNF141 |
| rs11042871 | 11:10531204-10531204 | RNF141 |
| rs118170201 | 11:10559890-10559890 | LYVE1 |
| rs117479720 | 11:10573528-10573528 | LYVE1 |
| rs117735090 | 11:16316761-16316761 | SOX6 |
| rs71486884 | 11:18867847-18867847 | MRGPRX7P |
| rs71486893 | 11:19050438-19050438 | MRGPRX2 |
| rs11038679 | 11:45837836-45837836 | - |
| rs139014611 | 11:47368136-47368136 | SPI1 |
| rs3911022 | 11:58019480-58019480 | OR9Q1 |
| rs66573492 | 11:58416324-58416324 | OR5B2 |
| rs1938602 | 11:58648003-58648003 | GLYAT |
| rs17494956 | 11:66224968-66224968 | PACS1 |
| rs12283611 | 11:83776234-83776234 | DLG2 |
| rs12290852 | 11:83776846-83776846 | DLG2 |
| rs74527019 | 11:97638101-97638101 | - |
| rs2509049 | 11:119095811-119095811 | DPAGT1 |
| rs7104278 | 11:129386409-129386409 | BARX2 |
| rs7111235 | 11:133788766-133788766 | LINC02743 |
| rs10791323 | 11:133832755-133832755 | - |
| rs73028346 | 11:133853489-133853489 | - |
| rs10750547 | 11:133859354-133859354 | - |

| | | |
|---|---|---|
| rs4937849 | 11:133887896-133887896 | - |
| rs329652 | 11:133899804-133899804 | IGSF9B |
| rs12800905 | 11:133919097-133919097 | IGSF9B |
| rs11223625 | 11:133926781-133926781 | IGSF9B |
| rs1793680 | 11:133928867-133928867 | IGSF9B |
| rs11223627 | 11:133930874-133930874 | IGSF9B |
| rs55880610 | 11:133977448-133977448 | - |
| rs4936216 | 11:133983113-133983113 | - |
| rs12273396 | 11:133986148-133986148 | - |
| rs148287061 | 12:21588466-21588466 | GYS2 |
| rs11047823 | 12:25090336-25090336 | LRMP |
| rs10843831 | 12:30742317-30742317 | CAPRIN2 |
| rs77669894 | 12:32089513-32089513 | - |
| rs189221759 | 12:32300735-32300735 | BICD1 |
| rs140427697 | 12:32338739-32338739 | BICD1 |
| rs146492306 | 12:33108774-33108774 | - |
| rs117154291 | 12:33403716-33403716 | SYT10 |
| rs190651674 | 12:34044984-34044984 | AC046130.2 |
| rs115468831 | 12:39617722-39617722 | ABCD2 |
| rs10784274 | 12:39909035-39909035 | SLC2A13 |
| rs7970063 | 12:39920244-39920244 | SLC2A13 |
| rs1497046 | 12:39928174-39928174 | SLC2A13 |
| rs12423983 | 12:39947809-39947809 | SLC2A13 |
| rs7962710 | 12:39970300-39970300 | SLC2A13 |
| rs12824918 | 12:39984971-39984971 | SLC2A13 |
| rs530602 | 12:39986233-39986233 | SLC2A13 |
| rs555740 | 12:39988897-39988897 | SLC2A13 |
| rs140722239 | 12:39994307-39994307 | SLC2A13 |
| rs17442108 | 12:40036632-40036632 | SLC2A13 |
| rs17442143 | 12:40044167-40044167 | SLC2A13 |
| rs1813122 | 12:40100944-40100944 | SLC2A13 |
| rs17458501 | 12:40102868-40102868 | SLC2A13 |
| rs79179868 | 12:40164911-40164911 | LINC02471 |
| rs2404577 | 12:40183917-40183917 | LRRK2-DT |
| rs11175546 | 12:40194013-40194013 | LRRK2-DT |
| rs2046931 | 12:40195305-40195305 | LRRK2-DT |
| rs79410089 | 12:40198262-40198262 | LRRK2-DT |
| rs7970326 | 12:40214089-40214089 | LRRK2-DT |
| rs76904798 | 12:40220632-40220632 | LRRK2 |
| rs1388598 | 12:40222364-40222364 | LRRK2 |
| rs17465751 | 12:40227079-40227079 | LRRK2 |
| rs4293189 | 12:40263423-40263423 | LRRK2 |
| rs111341533 | 12:40266936-40266936 | LRRK2 |
| rs17466339 | 12:40318557-40318557 | LRRK2 |
| rs11564147 | 12:40345691-40345691 | LRRK2 |
| rs11176261 | 12:40372450-40372450 | LRRK2 |

| | | |
|---|---|---|
| rs1019709 | 12:40408348-40408348 | MUC19 |
| rs2052802 | 12:40430937-40430937 | MUC19 |
| rs117774158 | 12:40432881-40432881 | MUC19 |
| rs11564228 | 12:40443529-40443529 | MUC19 |
| rs17128233 | 12:40458733-40458733 | MUC19 |
| rs11177373 | 12:40595381-40595381 | - |
| rs7960771 | 12:40607270-40607270 | - |
| rs144296031 | 12:40608183-40608183 | - |
| rs7134773 | 12:40648982-40648982 | - |
| rs66951529 | 12:40777688-40777688 | CNTN1 |
| rs1838343 | 12:40802428-40802428 | CNTN1 |
| rs10879497 | 12:40998221-40998221 | CNTN1 |
| rs144029582 | 12:41754811-41754811 | - |
| rs146006335 | 12:42925453-42925453 | AC068802.1 |
| rs2131709 | 12:44500625-44500625 | AC025253.1 |
| rs10880855 | 12:45751072-45751072 | ARID2 |
| rs1896539 | 12:46009539-46009539 | AC084878.1 |
| rs2408459 | 12:46047757-46047757 | - |
| rs10876470 | 12:53629707-53629707 | ATF7 |
| rs71465151 | 12:62379067-62379067 | USP15 |
| rs17661390 | 12:62452666-62452666 | - |
| rs118024659 | 12:63868716-63868716 | SRGAP1 |
| rs10777819 | 12:96903139-96903139 | NEDD1 |
| rs73168309 | 12:101806240-101806240 | GNPTAB |
| rs17032033 | 12:101893012-101893012 | DRAM1 |
| rs140811937 | 12:101925840-101925840 | DRAM1 |
| rs17038460 | 12:106454636-106454636 | POLR3B |
| rs4964178 | 12:106532721-106532721 | AC079385.1 |
| rs11113811 | 12:108315135-108315135 | CMKLR1 |
| rs10850060 | 12:109345885-109345885 | MYO1H |
| rs10774803 | 12:109620415-109620415 | - |
| rs6489158 | 12:122625818-122625818 | KNTC1 |
| rs34773022 | 12:122638867-122638867 | AC026333.4 |
| rs76554679 | 12:122655006-122655006 | - |
| rs9788287 | 12:122670716-122670716 | - |
| rs7313367 | 12:122727689-122727689 | HCAR1 |
| rs11059650 | 12:122735238-122735238 | HCAR1 |
| rs2245611 | 12:122738783-122738783 | - |
| rs193140864 | 12:122807291-122807291 | CCDC62 |
| rs12817488 | 12:122811747-122811747 | CCDC62 |
| rs11060344 | 12:122844554-122844554 | HIP1R |
| rs112484459 | 12:123003852-123003852 | PITPNM2 |
| rs185284989 | 12:123255582-123255582 | C12orf65 |
| rs28659953 | 12:123427666-123427666 | RILPL2 |
| rs113962677 | 12:130435916-130435916 | RIMBP2 |
| rs4418881 | 12:132481480-132481480 | - |

| | | |
|---|---|---|
| rs12811407 | 12:132493112-132493112 | FBRSL1 |
| rs10781619 | 12:132561748-132561748 | FBRSL1 |
| rs9507040 | 13:23166769-23166769 | LINC00362 |
| rs4769830 | 13:30402601-30402601 | AL161893.1 |
| rs9544930 | 13:35662291-35662291 | NBEA |
| rs9575700 | 13:36263862-36263862 | CCDC169 |
| rs1198502 | 13:49344829-49344829 | CAB39L |
| rs9535211 | 13:49365767-49365767 | CAB39L |
| rs9535230 | 13:49406382-49406382 | CAB39L |
| rs1127021 | 13:49721979-49721979 | KPNA3 |
| rs9584473 | 13:97034030-97034030 | - |
| rs7324681 | 13:97111528-97111528 | - |
| rs4771268 | 13:97212767-97212767 | - |
| rs6491345 | 13:97263104-97263104 | MBNL2 |
| rs2389911 | 13:97487423-97487423 | - |
| rs1805097 | 13:109782884-109782884 | IRS2 |
| rs9515121 | 13:109786818-109786818 | IRS2 |
| rs117146448 | 13:111206492-111206492 | ARHGEF7 |
| rs3858827 | 13:111242333-111242333 | ARHGEF7 |
| rs11625412 | 14:21417638-21417638 | CHD8 |
| rs61988340 | 14:37280387-37280387 | MIPOL1 |
| rs10133391 | 14:37536636-37536636 | MIPOL1 |
| rs10144632 | 14:54775618-54775618 | SAMD4A |
| rs709939 | 14:54782627-54782627 | SAMD4A |
| rs9323272 | 14:54822206-54822206 | - |
| rs55985131 | 14:54823301-54823301 | - |
| rs72715546 | 14:54918780-54918780 | - |
| rs72715548 | 14:54923307-54923307 | - |
| rs56206440 | 14:54984168-54984168 | WDHD1 |
| rs74617736 | 14:55154201-55154201 | DLGAP5 |
| rs6573020 | 14:55404734-55404734 | ATG14 |
| rs946065 | 14:55466201-55466201 | - |
| rs12880964 | 14:55495177-55495177 | KTN1-AS1 |
| rs1188074 | 14:55505113-55505113 | KTN1-AS1 |
| rs8010372 | 14:55551927-55551927 | KTN1-AS1 |
| rs12434554 | 14:55707387-55707387 | - |
| rs1959088 | 14:55731160-55731160 | - |
| rs1954483 | 14:62066382-62066382 | AL390816.1 |
| rs2681735 | 14:72345817-72345817 | RGS6 |
| rs888418 | 14:74652593-74652593 | AREL1 |
| rs2193597 | 14:74767626-74767626 | YLPM1 |
| rs917833 | 14:74912450-74912450 | RPS6KL1 |
| rs119512 | 14:87923196-87923196 | GALC |
| rs406280 | 14:87927161-87927161 | GALC |
| rs8005172 | 14:88006268-88006268 | GPR65 |
| rs8019631 | 14:88054358-88054358 | LINC01146 |

| | | |
|---|---|---|
| rs4900456 | 14:100145843-100145843 | DEGS2 |
| rs8008884 | 14:100187450-100187450 | - |
| rs118122626 | 14:100735293-100735293 | DLK1 |
| rs142070567 | 15:29469344-29469344 | FAM189A1 |
| rs10851405 | 15:41516888-41516888 | LTK |
| rs11635790 | 15:41588265-41588265 | TYRO3 |
| rs117618307 | 15:41738651-41738651 | MGA |
| rs117549128 | 15:48705628-48705628 | - |
| rs3098172 | 15:50480355-50480355 | USP8 |
| rs9635336 | 15:50506819-50506819 | USP8 |
| rs2250980 | 15:61704782-61704782 | AC018618.1 |
| rs11854497 | 15:61749234-61749234 | - |
| rs11071726 | 15:63098423-63098423 | AC087612.1 |
| rs116887089 | 15:97959417-97959417 | ARRDC4 |
| rs61747226 | 15:97969201-97969201 | ARRDC4 |
| rs1491184 | 15:97993366-97993366 | AC024651.1 |
| rs28385404 | 15:98034365-98034365 | AC022523.1 |
| rs12919274 | 16:1490005-1490005 | TELO2 |
| rs238680 | 16:1822159-1822159 | FAHD1 |
| rs72766631 | 16:2001427-2001427 | ZNF598 |
| rs8051877 | 16:2012064-2012064 | ZNF598 |
| rs26828 | 16:2194530-2194530 | CASKIN1 |
| rs1875206 | 16:9965150-9965150 | GRIN2A |
| rs12444013 | 16:11614990-11614990 | LITAF |
| rs2352795 | 16:19254563-19254563 | SYT17 |
| rs4072402 | 16:28925938-28925938 | RABEP2 |
| rs35695082 | 16:30781068-30781068 | ZNF629 |
| rs9939286 | 16:31150982-31150982 | PRSS36 |
| rs1548914 | 16:50445460-50445460 | AC007493.1 |
| rs1861761 | 16:50811622-50811622 | LINC02168 |
| rs8051542 | 16:52500255-52500255 | TOX3 |
| rs4784227 | 16:52565276-52565276 | CASC16 |
| rs3104783 | 16:52602330-52602330 | CASC16 |
| rs8059703 | 16:52922474-52922474 | - |
| rs10221156 | 16:52935514-52935514 | PHBP21 |
| rs8050560 | 16:52937867-52937867 | PHBP21 |
| rs74685800 | 16:52959266-52959266 | - |
| rs77419902 | 16:52960811-52960811 | - |
| rs11648976 | 16:53018339-53018339 | - |
| rs3934986 | 16:81867991-81867991 | PLCG2 |
| rs3102347 | 16:89303461-89303461 | ANKRD11 |
| rs4785677 | 16:89488693-89488693 | ANKRD11 |
| rs12600861 | 17:7452302-7452302 | CHRNB1 |
| rs6503037 | 17:7534733-7534733 | AC016876.1 |
| rs62059800 | 17:7545256-7545256 | TNFSF12 |
| rs190937141 | 17:7867619-7867619 | NAA38 |

| | | |
|---|---|---|
| rs12950912 | 17:7942329-7942329 | CNTROB |
| rs2278635 | 17:8087597-8087597 | MIR4314 |
| rs55844265 | 17:16127463-16127463 | NCOR1 |
| rs2245737 | 17:18241406-18241406 | LLGL1 |
| rs62070807 | 17:29570113-29570113 | GIT1 |
| rs146420943 | 17:31597422-31597422 | AC007923.3 |
| rs56171152 | 17:38740498-38740498 | PCGF2 |
| rs8070757 | 17:42448301-42448301 | - |
| rs12951632 | 17:42588995-42588995 | RETREG3 |
| rs62078933 | 17:44155037-44155037 | HROB |
| rs58392387 | 17:44220521-44220521 | UBTF |
| rs62078946 | 17:44244380-44244380 | SLC4A1 |
| rs850713 | 17:44350364-44350364 | GRN |
| rs35941271 | 17:44358852-44358852 | FAM171A2 |
| rs5910 | 17:44372421-44372421 | ITGA2B |
| rs113575323 | 17:44394009-44394009 | ITGA2B |
| rs59601921 | 17:44423044-44423044 | GPATCH8 |
| rs4793119 | 17:44547036-44547036 | - |
| rs4792956 | 17:44795863-44795863 | GJC1 |
| rs17544947 | 17:44841598-44841598 | HIGD1B |
| rs17629022 | 17:44914681-44914681 | GFAP |
| rs11652522 | 17:44978211-44978211 | AC015936.2 |
| rs1053578 | 17:45143918-45143918 | ACBD4 |
| rs72832877 | 17:45201209-45201209 | AC008105.3 |
| rs721579 | 17:45293115-45293115 | MAP3K14 |
| rs17686238 | 17:45339907-45339907 | - |
| rs9303471 | 17:45378976-45378976 | - |
| rs9895436 | 17:45380520-45380520 | - |
| rs117311539 | 17:45384761-45384761 | - |
| rs8327 | 17:45395141-45395141 | ARHGAP27 |
| rs12939187 | 17:45430857-45430857 | ARHGAP27 |
| rs8075717 | 17:45456956-45456956 | PLEKHM1 |
| rs62065439 | 17:45482589-45482589 | PLEKHM1 |
| rs1635303 | 17:45633467-45633467 | LINC02210 |
| rs118084908 | 17:45645238-45645238 | LINC02210 |
| rs62053943 | 17:45666837-45666837 | LINC02210-CRHR1 |
| rs143699161 | 17:45671346-45671346 | LINC02210-CRHR1 |
| rs1635287 | 17:45686474-45686474 | LINC02210-CRHR1 |
| rs1724425 | 17:45704381-45704381 | LINC02210-CRHR1 |
| rs62056907 | 17:45705809-45705809 | LINC02210-CRHR1 |
| rs1724386 | 17:45706394-45706394 | LINC02210-CRHR1 |

| | | |
|---|---|---|
| rs9912362 | 17:45706862-45706862 | LINC02210-CRHR1 |
| rs1880748 | 17:45711430-45711430 | LINC02210-CRHR1 |
| rs117615688 | 17:45720942-45720942 | LINC02210-CRHR1 |
| rs62054441 | 17:45751383-45751383 | LINC02210-CRHR1 |
| rs17335035 | 17:45752192-45752192 | LINC02210-CRHR1 |
| rs67492300 | 17:45760411-45760411 | LINC02210-CRHR1 |
| rs12940065 | 17:45788505-45788505 | CRHR1 |
| rs62057097 | 17:45795918-45795918 | CRHR1 |
| rs28364026 | 17:45834928-45834928 | CRHR1 |
| rs7221167 | 17:45855941-45855941 | MAPT-AS1 |
| rs2301689 | 17:45858472-45858472 | MAPT-AS1 |
| rs3785878 | 17:45896781-45896781 | MAPT |
| rs4792893 | 17:45908357-45908357 | MAPT |
| rs3785880 | 17:45916010-45916010 | MAPT |
| rs35908989 | 17:45916655-45916655 | MAPT |
| rs7210219 | 17:45941153-45941153 | MAPT |
| rs242557 | 17:45942346-45942346 | MAPT |
| rs3785885 | 17:45981274-45981274 | MAPT |
| rs2435207 | 17:45981562-45981562 | MAPT |
| rs713522 | 17:45987897-45987897 | MAPT |
| rs60969130 | 17:46013413-46013413 | MAPT |
| rs118185397 | 17:46063857-46063857 | KANSL1 |
| rs11652924 | 17:46104901-46104901 | KANSL1 |
| rs2668692 | 17:46215654-46215654 | AC217773.1 |
| rs35732828 | 17:46755851-46755851 | NSF |
| rs1563304 | 17:46797087-46797087 | WNT3 |
| rs3851781 | 17:46813935-46813935 | WNT3 |
| rs8069437 | 17:46829583-46829583 | WNT3 |
| rs58287249 | 17:46846427-46846427 | WNT9B |
| rs57986961 | 17:46870455-46870455 | WNT9B |
| rs28605133 | 17:50735379-50735379 | LUC7L3 |
| rs8066773 | 17:58524191-58524191 | SEPTIN4 |
| rs117987711 | 17:59033908-59033908 | TRIM37 |
| rs61169879 | 17:61840005-61840005 | BRIP1 |
| rs2278813 | 17:61962634-61962634 | MED13 |
| rs6416935 | 17:62107553-62107553 | - |
| rs59229385 | 17:62117416-62117416 | AC008158.1 |
| rs117639668 | 17:62144541-62144541 | POLRMTP1 |
| rs3088093 | 17:64129078-64129078 | ERN1 |
| rs7219674 | 17:78394116-78394116 | PGS1 |
| rs1623869 | 17:78429993-78429993 | DNAH17 |

| | | |
|---|---|---|
| rs72634334 | 17:82097050-82097050 | FASN |
| rs2123311 | 18:6403286-6403286 | L3MBTL4 |
| rs7237528 | 18:33630168-33630168 | ASXL3 |
| rs78550341 | 18:33646891-33646891 | ASXL3 |
| rs10775455 | 18:33730797-33730797 | ASXL3 |
| rs17754023 | 18:33843083-33843083 | - |
| rs16965005 | 18:33874080-33874080 | NOL4 |
| rs72963300 | 18:34437451-34437451 | - |
| rs75251687 | 18:42946139-42946139 | RIT2 |
| rs117096853 | 18:43016306-43016306 | RIT2 |
| rs4890245 | 18:43019205-43019205 | RIT2 |
| rs4890426 | 18:43092453-43092453 | RIT2 |
| rs72910682 | 18:43093026-43093026 | RIT2 |
| rs12456492 | 18:43093415-43093415 | RIT2 |
| rs56384227 | 18:43111033-43111033 | RIT2 |
| rs7241603 | 18:43217696-43217696 | - |
| rs7236839 | 18:43239306-43239306 | - |
| rs1549316 | 18:43258288-43258288 | - |
| rs111987322 | 18:47191759-47191759 | - |
| rs4940059 | 18:51182734-51182734 | MEX3C |
| rs1893378 | 18:51192490-51192490 | MEX3C |
| rs982983 | 18:52504938-52504938 | DCC |
| rs28634172 | 18:58822814-58822814 | AC104365.1 |
| rs3891810 | 18:69111089-69111089 | - |
| rs62091919 | 18:70283206-70283206 | - |
| rs10409758 | 19:1835953-1835953 | REXO1 |
| rs55818311 | 19:2341049-2341049 | SPPL2B |
| rs757322 | 19:2360856-2360856 | TMPRSS9 |
| rs12986109 | 19:2360969-2360969 | TMPRSS9 |
| rs72987495 | 19:2366244-2366244 | TMPRSS9 |
| rs11883051 | 19:6284444-6284444 | MLLT1 |
| rs12979469 | 19:10619676-10619676 | SLC44A2 |
| rs79277649 | 19:31529094-31529094 | LINC02841 |
| rs6509231 | 19:45655035-45655035 | RN7SL836P |
| rs4802553 | 19:49089723-49089723 | SNRNP70 |
| rs4801935 | 19:52775042-52775042 | ZNF600 |
| rs59858465 | 19:56401627-56401627 | ZNF583 |
| rs141547530 | 2:3188750-3188750 | EIPR1 |
| rs66504853 | 2:18103688-18103688 | KCNS3 |
| rs13387530 | 2:23720859-23720859 | - |
| rs72780106 | 2:23761964-23761964 | ATAD2B |
| rs34686340 | 2:24019080-24019080 | MFSD2B |
| rs56392651 | 2:24032338-24032338 | WDCP |
| rs4666140 | 2:28926185-28926185 | WDR43 |
| rs10182170 | 2:32479118-32479118 | BIRC6 |
| rs74818005 | 2:33653613-33653613 | AC017050.1 |

| | | |
|---|---|---|
| rs139870826 | 2:50817985-50817985 | NRXN1 |
| rs9309337 | 2:61536072-61536072 | XPO1 |
| rs34298249 | 2:62489836-62489836 | RN7SL18P |
| rs62178016 | 2:62507763-62507763 | TMEM17 |
| rs13416004 | 2:69437844-69437844 | NFU1 |
| rs4852283 | 2:69567218-69567218 | AAK1 |
| rs60806696 | 2:94889836-94889836 | AC097374.1 |
| rs116778080 | 2:95035241-95035241 | MAL |
| rs3105099 | 2:95041774-95041774 | MAL |
| rs3772044 | 2:95300298-95300298 | KCNIP3 |
| rs112032343 | 2:95378266-95378266 | KCNIP3 |
| rs6734326 | 2:96903998-96903998 | FAM178B |
| rs75094400 | 2:101525580-101525580 | PRCPP1 |
| rs13013794 | 2:101670030-101670030 | - |
| rs7590913 | 2:101689491-101689491 | - |
| rs62155725 | 2:101735899-101735899 | MAP4K4 |
| rs2298942 | 2:101852162-101852162 | MAP4K4 |
| rs10865049 | 2:101954596-101954596 | - |
| rs35789178 | 2:101987463-101987463 | IL1R2 |
| rs13020571 | 2:102042170-102042170 | AC007271.1 |
| rs61080885 | 2:102050083-102050083 | AC007271.1 |
| rs144201388 | 2:107997366-107997366 | SLC5A7 |
| rs1219690 | 2:122487007-122487007 | AC011246.1 |
| rs62166133 | 2:133810032-133810032 | - |
| rs115570081 | 2:134682340-134682340 | TMEM163 |
| rs10928510 | 2:134686370-134686370 | TMEM163 |
| rs10445684 | 2:134818755-134818755 | CCNT2-AS1 |
| rs78793148 | 2:136023692-136023692 | DARS-AS1 |
| rs77734975 | 2:144750626-144750626 | TEX41 |
| rs150409445 | 2:145016457-145016457 | TEX41 |
| rs723681 | 2:147910940-147910940 | ACVR2A |
| rs1965812 | 2:157814786-157814786 | ACVR1 |
| rs4547489 | 2:160302714-160302714 | RBMS1 |
| rs58757862 | 2:160378941-160378941 | RBMS1 |
| rs7572202 | 2:160429040-160429040 | RBMS1 |
| rs353128 | 2:165282098-165282098 | SCN2A |
| rs6740895 | 2:165283510-165283510 | SCN2A |
| rs353139 | 2:165289717-165289717 | SCN2A |
| rs1816918 | 2:165322847-165322847 | SCN2A |
| rs75629463 | 2:166127885-166127885 | SCN1A |
| rs7589835 | 2:166216341-166216341 | SCN9A |
| rs115032219 | 2:166265206-166265206 | SCN9A |
| rs1900831 | 2:168122780-168122780 | STK39 |
| rs12995897 | 2:168217639-168217639 | STK39 |
| rs1517320 | 2:168225732-168225732 | STK39 |
| rs13385320 | 2:168229316-168229316 | STK39 |

| | | |
|---|---|---|
| rs10175790 | 2:168238236-168238236 | STK39 |
| rs77611446 | 2:168249236-168249236 | STK39 |
| rs1474055 | 2:168253884-168253884 | - |
| rs2724176 | 2:168285532-168285532 | - |
| rs78851383 | 2:168318782-168318782 | - |
| rs16855271 | 2:168319860-168319860 | - |
| rs141858735 | 2:179466193-179466193 | ZNF385B |
| rs262266 | 2:180012435-180012435 | - |
| rs58695784 | 2:180057221-180057221 | - |
| rs13397184 | 2:180961506-180961506 | - |
| rs4853505 | 2:190382596-190382596 | - |
| rs3811607 | 2:190435676-190435676 | MFSD6 |
| rs281787 | 2:199919515-199919515 | C2orf69 |
| rs28418905 | 2:208323118-208323118 | PIKFYVE |
| rs145042764 | 2:208330711-208330711 | PIKFYVE |
| rs2271543 | 2:218277768-218277768 | PNKD |
| rs13000510 | 2:222500376-222500376 | SGPP2 |
| rs13010621 | 2:231009411-231009411 | SPATA3 |
| rs6758225 | 2:236030768-236030768 | AGAP1 |
| rs35449298 | 2:236034722-236034722 | AGAP1 |
| rs551859 | 2:238463195-238463195 | - |
| rs7353549 | 20:793346-793346 | - |
| rs2295545 | 20:3184040-3184040 | - |
| rs6037501 | 20:3210559-3210559 | ITPA |
| rs141459119 | 20:4623150-4623150 | RPS4XP2 |
| rs116158058 | 20:4652752-4652752 | RPS4XP2 |
| rs112864829 | 20:4861106-4861106 | SLC23A2 |
| rs111593423 | 20:6027580-6027580 | CRLS1 |
| rs4813918 | 20:10157279-10157279 | SNAP25-AS1 |
| rs11698183 | 20:25994949-25994949 | FAM182A |
| rs78184009 | 20:31851317-31851317 | DUSP15 |
| rs6119287 | 20:32815400-32815400 | MAPRE1 |
| rs293727 | 20:33345258-33345258 | BPIFB9P |
| rs6060042 | 20:34773918-34773918 | NCOA6 |
| rs6073118 | 20:43518146-43518146 | L3MBTL1 |
| rs729997 | 20:59411230-59411230 | - |
| rs1736023 | 21:15440563-15440563 | AJ009632.2 |
| rs12626383 | 21:15462709-15462709 | AJ009632.2 |
| rs1688101 | 21:19347156-19347156 | RNU1-139P |
| rs2835700 | 21:37350372-37350372 | AP001437.2 |
| rs11700462 | 21:37445510-37445510 | DYRK1A |
| rs2248244 | 21:37480059-37480059 | DYRK1A |
| rs73220444 | 21:37544098-37544098 | - |
| rs8131708 | 21:40047976-40047976 | DSCAM |
| rs9305690 | 21:40058461-40058461 | DSCAM |
| rs2837413 | 21:40067182-40067182 | DSCAM |

| | | |
|---|---|---|
| rs10222092 | 21:40082331-40082331 | DSCAM |
| rs12482422 | 21:40605066-40605066 | DSCAM |
| rs56379273 | 21:44406603-44406603 | TRPM2 |
| rs5751062 | 22:41202049-41202049 | L3MBTL2 |
| rs9611522 | 22:41231920-41231920 | L3MBTL2 |
| rs7290404 | 22:41353792-41353792 | ZC3H7B |
| rs74667571 | 22:50348547-50348547 | PPP6R2 |
| rs17046610 | 3:7024398-7024398 | GRM7 |
| rs7652281 | 3:8764054-8764054 | OXTR |
| rs55889070 | 3:18039779-18039779 | TBC1D5 |
| rs1108439 | 3:18159827-18159827 | TBC1D5 |
| rs9860554 | 3:18233751-18233751 | TBC1D5 |
| rs73038319 | 3:18320267-18320267 | TBC1D5 |
| rs78702810 | 3:18421039-18421039 | SATB1 |
| rs13095117 | 3:18704008-18704008 | SATB1-AS1 |
| rs341839 | 3:21379929-21379929 | - |
| rs7617877 | 3:28664273-28664273 | RBMS3 |
| rs10764 | 3:33496538-33496538 | CLASP2 |
| rs6766294 | 3:33613646-33613646 | CLASP2 |
| rs17075283 | 3:43184728-43184728 | - |
| rs75228828 | 3:46071391-46071391 | - |
| rs1982510 | 3:47011447-47011447 | SETD2 |
| rs56273245 | 3:47273006-47273006 | KIF9 |
| rs76060013 | 3:47728436-47728436 | SMARCC1 |
| rs9862131 | 3:48160950-48160950 | CDC25A |
| rs3731510 | 3:48179619-48179619 | CDC25A |
| rs990211 | 3:48683607-48683607 | NCKIPSD |
| rs6808104 | 3:48730444-48730444 | IP6K2 |
| rs137997194 | 3:48787504-48787504 | PRKAR2A |
| rs144070260 | 3:48812596-48812596 | PRKAR2A |
| rs143756010 | 3:49274815-49274815 | USP4 |
| rs115435316 | 3:49530748-49530748 | DAG1 |
| rs35726701 | 3:49703462-49703462 | RNF123 |
| rs34365302 | 3:52373396-52373396 | DNAH1 |
| rs613519 | 3:52434924-52434924 | SEMA3G |
| rs13083798 | 3:52615732-52615732 | PBRM1 |
| rs6765687 | 3:52699090-52699090 | GLT8D1 |
| rs36051354 | 3:52951996-52951996 | SFMBT1 |
| rs2178563 | 3:54791743-54791743 | CACNA2D3 |
| rs114413724 | 3:56515937-56515937 | - |
| rs28404421 | 3:58306758-58306758 | RPP14 |
| rs13085519 | 3:63650920-63650920 | SNTN |
| rs139277004 | 3:85818345-85818345 | CADM2 |
| rs4928106 | 3:101044767-101044767 | - |
| rs35489993 | 3:121693029-121693029 | GOLGB1 |
| rs4676755 | 3:121813788-121813788 | IQCB1 |

| | | |
|---|---|---|
| rs112613540 | 3:122348040-122348040 | - |
| rs116555124 | 3:122458413-122458413 | KPNA1 |
| rs55961674 | 3:122478045-122478045 | KPNA1 |
| rs7650313 | 3:122852588-122852588 | SLC49A4 |
| rs62408931 | 3:137110076-137110076 | - |
| rs79329661 | 3:141569914-141569914 | RASA2 |
| rs12485508 | 3:151383958-151383958 | MED12L |
| rs3821665 | 3:151416028-151416028 | MED12L |
| rs1426385 | 3:152280264-152280264 | MBNL1 |
| rs7629065 | 3:152412201-152412201 | MBNL1 |
| rs2222275 | 3:155641090-155641090 | PLCH1 |
| rs115083552 | 3:156226229-156226229 | KCNAB1 |
| rs11717272 | 3:161092567-161092567 | B3GALNT1 |
| rs1450522 | 3:161359842-161359842 | SPTSSB |
| rs77931272 | 3:167967106-167967106 | - |
| rs1967027 | 3:176245762-176245762 | - |
| rs73177372 | 3:182946022-182946022 | DCUN1D1 |
| rs76291226 | 3:183007775-183007775 | - |
| rs10513789 | 3:183042285-183042285 | MCCC1 |
| rs574718 | 3:183130193-183130193 | LAMP3 |
| rs79447920 | 3:183134426-183134426 | LAMP3 |
| rs79155994 | 3:183144347-183144347 | LAMP3 |
| rs580116 | 3:183154658-183154658 | LAMP3 |
| rs13084610 | 3:183163009-183163009 | LAMP3 |
| rs151139359 | 3:183218620-183218620 | MCF2L2 |
| rs13066057 | 3:183224883-183224883 | MCF2L2 |
| rs73191724 | 3:187271636-187271636 | MASP1 |
| rs28743077 | 4:714453-714453 | PCGF3 |
| rs11728488 | 4:716711-716711 | PCGF3 |
| rs7376288 | 4:747954-747954 | PCGF3 |
| rs3755966 | 4:764656-764656 | PCGF3 |
| rs62294076 | 4:765675-765675 | PCGF3 |
| rs77943567 | 4:808356-808356 | CPLX1 |
| rs147786984 | 4:814396-814396 | CPLX1 |
| rs7376690 | 4:819938-819938 | CPLX1 |
| rs11727899 | 4:833797-833797 | AC139887.3 |
| rs11736866 | 4:837393-837393 | AC139887.3 |
| rs2306241 | 4:850247-850247 | GAK |
| rs150789675 | 4:853009-853009 | GAK |
| rs144026795 | 4:880113-880113 | GAK |
| rs2279187 | 4:881741-881741 | GAK |
| rs3775119 | 4:899924-899924 | GAK |
| rs3775115 | 4:903225-903225 | GAK |
| rs62297083 | 4:903640-903640 | GAK |
| rs6826191 | 4:903876-903876 | GAK |
| rs139951812 | 4:905058-905058 | GAK |

| | | |
|---|---|---|
| rs116272755 | 4:908016-908016 | GAK |
| rs11724439 | 4:916733-916733 | GAK |
| rs873786 | 4:931588-931588 | TMEM175 |
| rs34311866 | 4:958159-958159 | TMEM175 |
| rs4690326 | 4:959910-959910 | TMEM175 |
| rs111344337 | 4:973547-973547 | DGKQ |
| rs147250021 | 4:988557-988557 | IDUA |
| rs10471250 | 4:995925-995925 | IDUA |
| rs148959726 | 4:999600-999600 | IDUA |
| rs10018140 | 4:1007954-1007954 | IDUA |
| rs35654957 | 4:1016289-1016289 | FGFRL1 |
| rs73219733 | 4:1019846-1019846 | FGFRL1 |
| rs4450872 | 4:1030938-1030938 | FGFRL1 |
| rs35321695 | 4:1036991-1036991 | - |
| rs17742599 | 4:1318606-1318606 | MAEA |
| rs2071695 | 4:2899622-2899622 | ADD1 |
| rs3021131 | 4:2917248-2917248 | ADD1 |
| rs1269355 | 4:2960056-2960056 | NOP14 |
| rs79419636 | 4:14165572-14165572 | AC092546.1 |
| rs17470482 | 4:15352611-15352611 | C1QTNF7 |
| rs79867298 | 4:15582292-15582292 | CC2D2A |
| rs4270584 | 4:15599176-15599176 | CC2D2A |
| rs1786 | 4:15662830-15662830 | FBXL5 |
| rs60040560 | 4:15695843-15695843 | FAM200B |
| rs4631042 | 4:15710927-15710927 | BST1 |
| rs73224662 | 4:15714466-15714466 | BST1 |
| rs12502586 | 4:15724941-15724941 | BST1 |
| rs12649015 | 4:15731397-15731397 | BST1 |
| rs2286879 | 4:15739257-15739257 | AC005798.1 |
| rs115383994 | 4:15749428-15749428 | - |
| rs73226561 | 4:15794465-15794465 | CD38 |
| rs35852935 | 4:17989899-17989899 | LCORL |
| rs13125804 | 4:17996420-17996420 | LCORL |
| rs56182412 | 4:41286424-41286424 | - |
| rs6532189 | 4:76100280-76100280 | ART3 |
| rs4859424 | 4:76110199-76110199 | NUP54 |
| rs113464411 | 4:76163978-76163978 | SCARB2 |
| rs12512579 | 4:76189879-76189879 | SCARB2 |
| rs13111888 | 4:76210141-76210141 | SCARB2 |
| rs78737354 | 4:76215705-76215705 | SCARB2 |
| rs11531413 | 4:76227031-76227031 | FAM47E |
| rs72655564 | 4:76248723-76248723 | FAM47E |
| rs13117238 | 4:76252761-76252761 | FAM47E |
| rs13149410 | 4:76252919-76252919 | FAM47E |
| rs2904221 | 4:76260880-76260880 | FAM47E |
| rs6812193 | 4:76277833-76277833 | FAM47E |

| | | |
|---|---|---|
| rs1542096 | 4:76283347-76283347 | FAM47E |
| rs79438017 | 4:76340366-76340366 | CCDC158 |
| rs4859670 | 4:76418579-76418579 | CCDC158 |
| rs17001966 | 4:76447040-76447040 | SHROOM3 |
| rs1986734 | 4:76499631-76499631 | SHROOM3 |
| rs10004356 | 4:76543872-76543872 | SHROOM3 |
| rs17015458 | 4:89350743-89350743 | - |
| rs114762839 | 4:89447394-89447394 | AC093866.1 |
| rs113166402 | 4:89479010-89479010 | AC093866.1 |
| rs112744012 | 4:89491284-89491284 | AC093866.1 |
| rs11736388 | 4:89527919-89527919 | AC093866.1 |
| rs56958658 | 4:89528456-89528456 | AC093866.1 |
| rs67262058 | 4:89532090-89532090 | AC093866.1 |
| rs10018847 | 4:89578394-89578394 | AC093866.1 |
| rs12512230 | 4:89582452-89582452 | AC093866.1 |
| rs72655899 | 4:89617316-89617316 | AC093866.1 |
| rs116040410 | 4:89621988-89621988 | AC093866.1 |
| rs79208813 | 4:89624344-89624344 | AC093866.1 |
| rs79083944 | 4:89625335-89625335 | AC093866.1 |
| rs10027842 | 4:89630392-89630392 | AC093866.1 |
| rs13140923 | 4:89645318-89645318 | AC093866.1 |
| rs142824388 | 4:89648089-89648089 | AC093866.1 |
| rs356231 | 4:89652245-89652245 | AC093866.1 |
| rs72657707 | 4:89657360-89657360 | AC093866.1 |
| rs111763892 | 4:89661030-89661030 | AC093866.1 |
| rs145954321 | 4:89676494-89676494 | AC093866.1 |
| rs7697331 | 4:89679848-89679848 | AC093866.1 |
| rs9996301 | 4:89685485-89685485 | AC093866.1 |
| rs17286290 | 4:89697113-89697113 | AC093866.1 |
| rs71609573 | 4:89703442-89703442 | AC093866.1 |
| rs12505231 | 4:89704658-89704658 | AC093866.1 |
| rs356182 | 4:89704960-89704960 | AC093866.1 |
| rs71609574 | 4:89706816-89706816 | AC093866.1 |
| rs114685218 | 4:89710874-89710874 | AC093866.1 |
| rs62306284 | 4:89721380-89721380 | SNCA |
| rs17180453 | 4:89731983-89731983 | SNCA |
| rs35267877 | 4:89744512-89744512 | SNCA |
| rs1372518 | 4:89836143-89836143 | SNCA |
| rs116227916 | 4:89842088-89842088 | SNCA |
| rs111578789 | 4:89852969-89852969 | - |
| rs72657784 | 4:89867792-89867792 | - |
| rs7693616 | 4:89871079-89871079 | - |
| rs114828194 | 4:89883381-89883381 | MMRN1 |
| rs72657797 | 4:89899658-89899658 | MMRN1 |
| rs17806425 | 4:89914407-89914407 | MMRN1 |
| rs79214011 | 4:89938128-89938128 | MMRN1 |

| | | |
|---|---|---|
| rs145948000 | 4:89961392-89961392 | - |
| rs17016417 | 4:90001148-90001148 | AC105445.1 |
| rs139616478 | 4:90006087-90006087 | - |
| rs13107054 | 4:90061761-90061761 | - |
| rs73832568 | 4:90091011-90091011 | - |
| rs140962713 | 4:90103027-90103027 | - |
| rs17810668 | 4:90136643-90136643 | CCSER1 |
| rs116166250 | 4:90212111-90212111 | CCSER1 |
| rs77713265 | 4:90242889-90242889 | CCSER1 |
| rs77738892 | 4:90386840-90386840 | CCSER1 |
| rs78077825 | 4:90433339-90433339 | CCSER1 |
| rs116582845 | 4:90502748-90502748 | CCSER1 |
| rs17240838 | 4:90612466-90612466 | CCSER1 |
| rs13107325 | 4:102267552-102267552 | SLC39A8 |
| rs13117519 | 4:113447909-113447909 | CAMK2D |
| rs62314976 | 4:113464520-113464520 | CAMK2D |
| rs7689195 | 4:113477097-113477097 | CAMK2D |
| rs17046126 | 4:113510917-113510917 | CAMK2D |
| rs4834349 | 4:113526035-113526035 | CAMK2D |
| rs6821748 | 4:127907256-127907256 | - |
| rs9993435 | 4:128164348-128164348 | LARP1B |
| rs7687276 | 4:169398320-169398320 | NEK1 |
| rs17055075 | 4:169652145-169652145 | CLCN3 |
| rs62333164 | 4:169662006-169662006 | CLCN3 |
| rs34181295 | 4:188735497-188735497 | AC093909.1 |
| rs116699996 | 5:28167023-28167023 | - |
| rs3797237 | 5:54241912-54241912 | ARL15 |
| rs10074908 | 5:60538793-60538793 | PART1 |
| rs78348576 | 5:60548774-60548774 | PART1 |
| rs4700387 | 5:60740924-60740924 | KRT8P31 |
| rs78620886 | 5:60791509-60791509 | ELOVL7 |
| rs142805273 | 5:60831468-60831468 | ELOVL7 |
| rs139357177 | 5:60841561-60841561 | ELOVL7 |
| rs158927 | 5:60933439-60933439 | ERCC8 |
| rs78984066 | 5:60954235-60954235 | NDUFAF2 |
| rs76339698 | 5:61076761-61076761 | NDUFAF2 |
| rs256376 | 5:61186277-61186277 | SMIM15-AS1 |
| rs10052849 | 5:61238254-61238254 | LINC02057 |
| rs10471513 | 5:61307635-61307635 | LINC02057 |
| rs6453214 | 5:76288598-76288598 | SV2C |
| rs10060273 | 5:80322830-80322830 | SPZ1 |
| rs112216323 | 5:80352963-80352963 | AC026410.3 |
| rs13161403 | 5:88471144-88471144 | AC091826.3 |
| rs60470187 | 5:90756146-90756146 | ADGRV1 |
| rs7709726 | 5:102991759-102991759 | PAM |
| rs377192 | 5:103267853-103267853 | MACIR |

| | | |
|---|---|---|
| rs2561481 | 5:103358411-103358411 | - |
| rs10061674 | 5:103371008-103371008 | - |
| rs294068 | 5:103467697-103467697 | PDZPH1P |
| rs76801032 | 5:107836324-107836324 | - |
| rs28786120 | 5:120359407-120359407 | AC113418.1 |
| rs112723361 | 5:120490287-120490287 | PRR16 |
| rs4282308 | 5:124690450-124690450 | ZNF608 |
| rs11241766 | 5:124741569-124741569 | ZNF608 |
| rs6875262 | 5:124774580-124774580 | - |
| rs1078675 | 5:124881569-124881569 | LINC02240 |
| rs57750646 | 5:132344571-132344571 | SLC22A4 |
| rs77949585 | 5:134588788-134588788 | - |
| rs11950533 | 5:134863415-134863415 | C5orf24 |
| rs13361619 | 5:138312990-138312990 | CDC25C |
| rs3798153 | 5:138446515-138446515 | REEP2 |
| rs12657476 | 5:151186635-151186635 | CCDC69 |
| rs113516381 | 5:177030476-177030476 | ZNF346 |
| rs3846912 | 6:13656808-13656808 | RANBP9 |
| rs1204148 | 6:13817127-13817127 | MCUR1 |
| rs625964 | 6:20016736-20016736 | - |
| rs6909187 | 6:25785697-25785697 | SLC17A1 |
| rs62396221 | 6:26285520-26285520 | H4C8 |
| rs9379946 | 6:26968286-26968286 | LINC00240 |
| rs7764984 | 6:27066402-27066402 | - |
| rs7771953 | 6:27303564-27303564 | POM121L2 |
| rs2235233 | 6:27312073-27312073 | POM121L2 |
| rs9461358 | 6:27330051-27330051 | VN1R10P |
| rs9468076 | 6:27371111-27371111 | ZNF391 |
| rs6931659 | 6:27508813-27508813 | AL021918.2 |
| rs6936653 | 6:27597051-27597051 | RNU6-471P |
| rs17693877 | 6:27739267-27739267 | GPR89P |
| rs4140646 | 6:27771022-27771022 | - |
| rs149979 | 6:28023229-28023229 | ZSCAN16-AS1 |
| rs149957 | 6:28069242-28069242 | OR1F12 |
| rs17767294 | 6:28086420-28086420 | ZNF165 |
| rs11970409 | 6:28627330-28627330 | - |
| rs9378107 | 6:28793906-28793906 | - |
| rs3117434 | 6:29295973-29295973 | AL672167.1 |
| rs4947236 | 6:29755897-29755897 | IFITM4P |
| rs1611182 | 6:29813272-29813272 | MICG |
| rs3128908 | 6:29856490-29856490 | AL645929.3 |
| rs2517718 | 6:29948614-29948614 | HLA-A |
| rs2240068 | 6:30108853-30108853 | TRIM31 |
| rs9261439 | 6:30122560-30122560 | - |
| rs3132676 | 6:30148301-30148301 | TRIM40 |
| rs4493062 | 6:30248780-30248780 | HCG17 |

| | | |
|---|---|---|
| rs2016216 | 6:30410049-30410049 | MICC |
| rs4713327 | 6:30415991-30415991 | MICC |
| rs1265075 | 6:31145336-31145336 | CCHCR1 |
| rs9263850 | 6:31188411-31188411 | - |
| rs28397271 | 6:31196866-31196866 | HCG27 |
| rs6930643 | 6:31206258-31206258 | HCG27 |
| rs28744244 | 6:31209999-31209999 | - |
| rs2853923 | 6:31297960-31297960 | LINC02571 |
| rs6909636 | 6:31378822-31378822 | ZDHHC20P2 |
| rs3130062 | 6:31558135-31558135 | NFKBIL1 |
| rs3115668 | 6:31673708-31673708 | LY6G5C |
| rs494620 | 6:31870936-31870936 | SLC44A4 |
| rs1738434 | 6:31976259-31976259 | DXO |
| rs17208000 | 6:32142161-32142161 | - |
| rs3130346 | 6:32163733-32163733 | PPT2 |
| rs9267922 | 6:32238688-32238688 | - |
| rs2073044 | 6:32371209-32371209 | TSBP1 |
| rs9268402 | 6:32373576-32373576 | TSBP1 |
| rs9268499 | 6:32407918-32407918 | BTNL2 |
| rs9268500 | 6:32408740-32408740 | BTNL2 |
| rs3135359 | 6:32422801-32422801 | - |
| rs4321864 | 6:32431410-32431410 | - |
| rs9268652 | 6:32441279-32441279 | HLA-DRA |
| rs3177928 | 6:32444658-32444658 | HLA-DRA |
| rs3135384 | 6:32452182-32452182 | - |
| rs9268932 | 6:32465513-32465513 | HLA-DRB9 |
| rs9271149 | 6:32609678-32609678 | - |
| rs536810 | 6:32609720-32609720 | - |
| rs532965 | 6:32610196-32610196 | - |
| rs4959105 | 6:32615369-32615369 | - |
| rs9271375 | 6:32619290-32619290 | - |
| rs28383314 | 6:32619436-32619436 | - |
| rs9271500 | 6:32621523-32621523 | - |
| rs9275211 | 6:32690067-32690067 | - |
| rs2858329 | 6:32691024-32691024 | - |
| rs9275348 | 6:32699861-32699861 | - |
| rs1794275 | 6:32703471-32703471 | MTCO3P1 |
| rs9275546 | 6:32708668-32708668 | MTCO3P1 |
| rs2858331 | 6:32713500-32713500 | AL662789.1 |
| rs3873448 | 6:32715278-32715278 | AL662789.1 |
| rs9276434 | 6:32744889-32744889 | HLA-DQA2 |
| rs35805736 | 6:32794748-32794748 | - |
| rs6457681 | 6:32805720-32805720 | - |
| rs115360810 | 6:32828533-32828533 | TAP2 |
| rs206776 | 6:32985934-32985934 | BRD2 |
| rs206775 | 6:32986857-32986857 | - |

| | | |
|---|---|---|
| rs1442222 | 6:46246324-46246324 | RCAN2 |
| rs9446462 | 6:71747595-71747595 | - |
| rs10455248 | 6:71772755-71772755 | - |
| rs9442714 | 6:71779330-71779330 | - |
| rs9389810 | 6:99881570-99881570 | - |
| rs9481193 | 6:111810445-111810445 | FYN |
| rs57650567 | 6:111851753-111851753 | FYN |
| rs17073085 | 6:111947496-111947496 | - |
| rs36142021 | 6:118838319-118838319 | MCM9 |
| rs12206988 | 6:119085363-119085363 | FAM184A |
| rs6569038 | 6:119095307-119095307 | FAM184A |
| rs7769687 | 6:125912545-125912545 | NCOA7 |
| rs78243222 | 6:132726490-132726490 | VNN3 |
| rs75859381 | 6:132889222-132889222 | AL137783.1 |
| rs117701920 | 6:132987766-132987766 | LINC00326 |
| rs12662170 | 6:150925255-150925255 | MTHFD1L |
| rs9365002 | 6:158903689-158903689 | C6orf99 |
| rs77846331 | 6:159910464-159910464 | MAS1 |
| rs7744123 | 6:168219979-168219979 | AL606970.5 |
| rs73047368 | 7:2726762-2726762 | GNA12 |
| rs78391336 | 7:2793074-2793074 | GNA12 |
| rs113918424 | 7:6333933-6333933 | FAM220A |
| rs61247228 | 7:6612156-6612156 | C7orf26 |
| rs12702597 | 7:7272864-7272864 | AC005532.1 |
| rs2041361 | 7:22995675-22995675 | FAM126A |
| rs7782981 | 7:23064778-23064778 | - |
| rs78614415 | 7:23109624-23109624 | KLHL7 |
| rs77551378 | 7:23178069-23178069 | NUP42 |
| rs1728313 | 7:23231412-23231412 | GPNMB |
| rs34323745 | 7:23272449-23272449 | GPNMB |
| rs1964536 | 7:23340566-23340566 | IGF2BP3 |
| rs62468198 | 7:23343361-23343361 | IGF2BP3 |
| rs10488282 | 7:23343516-23343516 | IGF2BP3 |
| rs35620312 | 7:23387177-23387177 | IGF2BP3 |
| rs111665084 | 7:23388101-23388101 | IGF2BP3 |
| rs377383 | 7:23395434-23395434 | IGF2BP3 |
| rs188081801 | 7:23564549-23564549 | AC006026.1 |
| rs140622649 | 7:36358639-36358639 | KIAA0895 |
| rs78241966 | 7:36446251-36446251 | ANLN |
| rs41279603 | 7:43943193-43943193 | UBE2D4 |
| rs62469301 | 7:65202049-65202049 | INTS4P1 |
| rs118157596 | 7:65908978-65908978 | VKORC1L1 |
| rs12540307 | 7:66197780-66197780 | - |
| rs62465432 | 7:66536369-66536369 | AC008267.3 |
| rs17138017 | 7:66927805-66927805 | TMEM248 |
| rs62466588 | 7:66993916-66993916 | SBDS |

| | | |
|---|---|---|
| rs1075007 | 7:78481252-78481252 | MAGI2 |
| rs62461391 | 7:82854172-82854172 | PCLO |
| rs76083843 | 7:89304380-89304380 | ZNF804B |
| rs10215799 | 7:94675032-94675032 | - |
| rs17166875 | 7:95411959-95411959 | PON2 |
| rs2237585 | 7:95420506-95420506 | PON2 |
| rs4595055 | 7:95707291-95707291 | - |
| rs6979335 | 7:100492237-100492237 | NYAP1 |
| rs7805498 | 7:100537297-100537297 | AGFG2 |
| rs4599742 | 7:106050650-106050650 | - |
| rs76305012 | 7:117557301-117557301 | CFTR |
| rs79006095 | 7:120333784-120333784 | KCND2 |
| rs73166011 | 7:149436878-149436878 | ZNF777 |
| rs1971411 | 8:8415897-8415897 | AC103957.1 |
| rs2921073 | 8:8450133-8450133 | - |
| rs17153607 | 8:11657684-11657684 | - |
| rs117059721 | 8:11704908-11704908 | GATA4 |
| rs11785481 | 8:11759633-11759633 | GATA4 |
| rs2686197 | 8:11804411-11804411 | FDFT1 |
| rs1736060 | 8:11807229-11807229 | FDFT1 |
| rs80040100 | 8:11812167-11812167 | FDFT1 |
| rs3735810 | 8:11835332-11835332 | FDFT1 |
| rs6990033 | 8:11835869-11835869 | FDFT1 |
| rs62495697 | 8:11851498-11851498 | CTSB |
| rs73209041 | 8:11857432-11857432 | CTSB |
| rs589518 | 8:16839151-16839151 | AC011586.2 |
| rs576703 | 8:16846568-16846568 | AC011586.2 |
| rs823425 | 8:16857814-16857814 | AC011586.2 |
| rs34937419 | 8:16859081-16859081 | AC011586.2 |
| rs1960437 | 8:16873245-16873245 | AC011586.2 |
| rs62492658 | 8:22627473-22627473 | BIN3 |
| rs4424255 | 8:22664616-22664616 | BIN3 |
| rs2280104 | 8:22668467-22668467 | BIN3 |
| rs1532278 | 8:27608798-27608798 | CLU |
| rs7837112 | 8:28907976-28907976 | HMBOX1 |
| rs7011522 | 8:35029242-35029242 | - |
| rs118027082 | 8:73952340-73952340 | ELOC |
| rs117582595 | 8:104624038-104624038 | ZFPM2 |
| rs12541194 | 8:104637830-104637830 | ZFPM2 |
| rs6469271 | 8:109632545-109632545 | SYBU |
| rs800902 | 8:115416489-115416489 | TRPS1 |
| rs73337990 | 8:124422412-124422412 | - |
| rs17372350 | 8:124576693-124576693 | MTSS1 |
| rs3857948 | 8:124598539-124598539 | MTSS1 |
| rs10103418 | 8:129886017-129886017 | CYRIB |
| rs2086641 | 8:129889663-129889663 | CYRIB |

| | | |
|---|---|---|
| rs298606 | 8:129927276-129927276 | CYRIB |
| rs1902015 | 8:130028693-130028693 | - |
| rs28396295 | 8:130383466-130383466 | ASAP1 |
| rs75393804 | 8:134506857-134506857 | ZFAT |
| rs75582926 | 8:134523293-134523293 | ZFAT |
| rs1060832 | 8:140531789-140531789 | AGO2 |
| rs149865921 | 8:141499890-141499890 | MROH5 |
| rs117511259 | 9:4064097-4064097 | GLIS3 |
| rs2754320 | 9:17540625-17540625 | - |
| rs116439081 | 9:17576064-17576064 | SH3GL2 |
| rs117244129 | 9:17577945-17577945 | SH3GL2 |
| rs10756891 | 9:17584276-17584276 | SH3GL2 |
| rs10963177 | 9:17639460-17639460 | SH3GL2 |
| rs2038521 | 9:17640024-17640024 | SH3GL2 |
| rs10810834 | 9:17713237-17713237 | SH3GL2 |
| rs3808689 | 9:17723232-17723232 | SH3GL2 |
| rs2209440 | 9:17736344-17736344 | SH3GL2 |
| rs75170096 | 9:17741228-17741228 | SH3GL2 |
| rs3824370 | 9:17758947-17758947 | SH3GL2 |
| rs117777538 | 9:17799676-17799676 | SH3GL2 |
| rs12236209 | 9:17828041-17828041 | - |
| rs78062691 | 9:32257417-32257417 | - |
| rs10122038 | 9:33911057-33911057 | UBE2R2 |
| rs2026658 | 9:34156814-34156814 | - |
| rs76639411 | 9:80868137-80868137 | AL138749.1 |
| rs2025943 | 9:89153960-89153960 | SHC3 |
| rs7862334 | 9:90493810-90493810 | LINC01501 |
| rs10821079 | 9:93161958-93161958 | - |
| rs13289468 | 9:125418053-125418053 | AL627223.2 |
| rs13283793 | 9:125679362-125679362 | MAPKAP1 |
| rs113253813 | 9:127498834-127498834 | LRSAM1 |

**Supplementary table 4: SNPs included in the 'best' PRS, after exclusion of all SNPs within a 1MB window of PD risk loci.. Annotations are given in hg38 and nearest genes are derived from Ensembl Variant Event Predictor.**

| RSID | CHR:BP (hg38) | Nearest gene |
|---|---|---|
| rs845250 | 1:7423291-7423291 | CAMTA1 |
| rs77901899 | 1:62271453-62271453 | KANK4 |
| rs148303184 | 1:62567432-62567432 | DOCK7 |
| rs4655643 | 1:66624416-66624416 | SGIP1 |
| rs11208947 | 1:66689781-66689781 | SGIP1 |
| rs278839 | 1:77642189-77642189 | AC118549.1 |
| rs11164880 | 1:93192539-93192539 | CCDC18 |
| rs114809584 | 1:96889283-96889283 | - |
| rs9728526 | 1:145718303-145718303 | CD160 |
| rs74916032 | 1:147535311-147535311 | - |
| rs201694776 | 1:152616206-152616206 | LCE3B |
| rs12748659 | 1:156330207-156330207 | CCT3 |
| rs12141455 | 1:193352957-193352957 | LINC01031 |
| rs55922528 | 1:194585600-194585600 | - |
| rs78203442 | 1:194766300-194766300 | - |
| rs116592662 | 1:194863221-194863221 | AL353072.2 |
| rs4915470 | 1:200965282-200965282 | KIF21B |
| rs296562 | 1:200976146-200976146 | KIF21B |
| rs55925257 | 1:227871638-227871638 | - |
| rs147393995 | 1:228039106-228039106 | WNT3A |
| rs34249947 | 1:228136466-228136466 | GUK1 |
| rs9428576 | 1:243498725-243498725 | AKT3 |
| rs12408455 | 1:243578639-243578639 | AKT3 |
| rs79305906 | 10:22679230-22679230 | PIP4K2A |
| rs2754493 | 10:38362521-38362521 | HSD17B7P2 |
| rs2489678 | 10:42458987-42458987 | CCNYL2 |
| rs11239219 | 10:44720664-44720664 | - |
| rs10857613 | 10:48621632-48621632 | ARHGAP22 |
| rs17721694 | 10:50407533-50407533 | SGMS1 |
| rs72809473 | 10:75695702-75695702 | LRMDA |
| rs846578 | 10:76739750-76739750 | - |
| rs111691636 | 10:77615783-77615783 | KCNMA1 |
| rs3781273 | 10:93643216-93643216 | PDE6C |
| rs142328600 | 10:94008085-94008085 | - |
| rs10882978 | 10:97663991-97663991 | PI4K2A |
| rs3740484 | 10:100987606-100987606 | SEMA4G |
| rs117542248 | 10:117751683-117751683 | LINC02674 |
| rs3117448 | 10:132351935-132351935 | LRRC27 |
| rs117011293 | 11:527311-527311 | HRAS |
| rs4963136 | 11:549119-549119 | LRRC56 |
| rs10835060 | 11:1466913-1466913 | BRSK2 |
| rs10840502 | 11:2179719-2179719 | - |

| | | |
|---|---|---|
| rs7933395 | 11:7508194-7508194 | OLFML1 |
| rs117735090 | 11:16316761-16316761 | SOX6 |
| rs71486884 | 11:18867847-18867847 | MRGPRX7P |
| rs71486893 | 11:19050438-19050438 | MRGPRX2 |
| rs11038679 | 11:45837836-45837836 | - |
| rs139014611 | 11:47368136-47368136 | SPI1 |
| rs3911022 | 11:58019480-58019480 | OR9Q1 |
| rs66573492 | 11:58416324-58416324 | OR5B2 |
| rs1938602 | 11:58648003-58648003 | GLYAT |
| rs17494956 | 11:66224968-66224968 | PACS1 |
| rs74527019 | 11:97638101-97638101 | - |
| rs2509049 | 11:119095811-119095811 | DPAGT1 |
| rs7104278 | 11:129386409-129386409 | BARX2 |
| rs148287061 | 12:21588466-21588466 | GYS2 |
| rs11047823 | 12:25090336-25090336 | LRMP |
| rs10843831 | 12:30742317-30742317 | CAPRIN2 |
| rs77669894 | 12:32089513-32089513 | - |
| rs189221759 | 12:32300735-32300735 | BICD1 |
| rs140427697 | 12:32338739-32338739 | BICD1 |
| rs146492306 | 12:33108774-33108774 | - |
| rs117154291 | 12:33403716-33403716 | SYT10 |
| rs190651674 | 12:34044984-34044984 | AC046130.2 |
| rs144029582 | 12:41754811-41754811 | - |
| rs146006335 | 12:42925453-42925453 | AC068802.1 |
| rs2131709 | 12:44500625-44500625 | AC025253.1 |
| rs10876470 | 12:53629707-53629707 | ATF7 |
| rs71465151 | 12:62379067-62379067 | USP15 |
| rs17661390 | 12:62452666-62452666 | - |
| rs118024659 | 12:63868716-63868716 | SRGAP1 |
| rs10777819 | 12:96903139-96903139 | NEDD1 |
| rs73168309 | 12:101806240-101806240 | GNPTAB |
| rs17032033 | 12:101893012-101893012 | DRAM1 |
| rs140811937 | 12:101925840-101925840 | DRAM1 |
| rs17038460 | 12:106454636-106454636 | POLR3B |
| rs4964178 | 12:106532721-106532721 | AC079385.1 |
| rs11113811 | 12:108315135-108315135 | CMKLR1 |
| rs10850060 | 12:109345885-109345885 | MYO1H |
| rs10774803 | 12:109620415-109620415 | - |
| rs113962677 | 12:130435916-130435916 | RIMBP2 |
| rs9507040 | 13:23166769-23166769 | LINC00362 |
| rs4769830 | 13:30402601-30402601 | AL161893.1 |
| rs9544930 | 13:35662291-35662291 | NBEA |
| rs9575700 | 13:36263862-36263862 | CCDC169 |
| rs1805097 | 13:109782884-109782884 | IRS2 |
| rs9515121 | 13:109786818-109786818 | IRS2 |
| rs117146448 | 13:111206492-111206492 | ARHGEF7 |

| | | |
|---|---|---|
| rs3858827 | 13:111242333-111242333 | ARHGEF7 |
| rs11625412 | 14:21417638-21417638 | CHD8 |
| rs1954483 | 14:62066382-62066382 | AL390816.1 |
| rs2681735 | 14:72345817-72345817 | RGS6 |
| rs4900456 | 14:100145843-100145843 | DEGS2 |
| rs8008884 | 14:100187450-100187450 | - |
| rs118122626 | 14:100735293-100735293 | DLK1 |
| rs142070567 | 15:29469344-29469344 | FAM189A1 |
| rs10851405 | 15:41516888-41516888 | LTK |
| rs11635790 | 15:41588265-41588265 | TYRO3 |
| rs117618307 | 15:41738651-41738651 | MGA |
| rs117549128 | 15:48705628-48705628 | - |
| rs3098172 | 15:50480355-50480355 | USP8 |
| rs9635336 | 15:50506819-50506819 | USP8 |
| rs11071726 | 15:63098423-63098423 | AC087612.1 |
| rs116887089 | 15:97959417-97959417 | ARRDC4 |
| rs61747226 | 15:97969201-97969201 | ARRDC4 |
| rs1491184 | 15:97993366-97993366 | AC024651.1 |
| rs28385404 | 15:98034365-98034365 | AC022523.1 |
| rs12919274 | 16:1490005-1490005 | TELO2 |
| rs238680 | 16:1822159-1822159 | FAHD1 |
| rs72766631 | 16:2001427-2001427 | ZNF598 |
| rs8051877 | 16:2012064-2012064 | ZNF598 |
| rs26828 | 16:2194530-2194530 | CASKIN1 |
| rs1875206 | 16:9965150-9965150 | GRIN2A |
| rs12444013 | 16:11614990-11614990 | LITAF |
| rs3934986 | 16:81867991-81867991 | PLCG2 |
| rs3102347 | 16:89303461-89303461 | ANKRD11 |
| rs4785677 | 16:89488693-89488693 | ANKRD11 |
| rs55844265 | 17:16127463-16127463 | NCOR1 |
| rs2245737 | 17:18241406-18241406 | LLGL1 |
| rs62070807 | 17:29570113-29570113 | GIT1 |
| rs146420943 | 17:31597422-31597422 | AC007923.3 |
| rs56171152 | 17:38740498-38740498 | PCGF2 |
| rs28605133 | 17:50735379-50735379 | LUC7L3 |
| rs8066773 | 17:58524191-58524191 | SEPTIN4 |
| rs117987711 | 17:59033908-59033908 | TRIM37 |
| rs3088093 | 17:64129078-64129078 | ERN1 |
| rs72634334 | 17:82097050-82097050 | FASN |
| rs2123311 | 18:6403286-6403286 | L3MBTL4 |
| rs111987322 | 18:47191759-47191759 | - |
| rs982983 | 18:52504938-52504938 | DCC |
| rs28634172 | 18:58822814-58822814 | AC104365.1 |
| rs3891810 | 18:69111089-69111089 | - |
| rs62091919 | 18:70283206-70283206 | - |
| rs11883051 | 19:6284444-6284444 | MLLT1 |

| | | |
|---|---|---|
| rs12979469 | 19:10619676-10619676 | SLC44A2 |
| rs79277649 | 19:31529094-31529094 | LINC02841 |
| rs6509231 | 19:45655035-45655035 | RN7SL836P |
| rs4802553 | 19:49089723-49089723 | SNRNP70 |
| rs4801935 | 19:52775042-52775042 | ZNF600 |
| rs59858465 | 19:56401627-56401627 | ZNF583 |
| rs141547530 | 2:3188750-3188750 | EIPR1 |
| rs13387530 | 2:23720859-23720859 | - |
| rs72780106 | 2:23761964-23761964 | ATAD2B |
| rs34686340 | 2:24019080-24019080 | MFSD2B |
| rs56392651 | 2:24032338-24032338 | WDCP |
| rs4666140 | 2:28926185-28926185 | WDR43 |
| rs10182170 | 2:32479118-32479118 | BIRC6 |
| rs74818005 | 2:33653613-33653613 | AC017050.1 |
| rs139870826 | 2:50817985-50817985 | NRXN1 |
| rs9309337 | 2:61536072-61536072 | XPO1 |
| rs34298249 | 2:62489836-62489836 | RN7SL18P |
| rs62178016 | 2:62507763-62507763 | TMEM17 |
| rs13416004 | 2:69437844-69437844 | NFU1 |
| rs4852283 | 2:69567218-69567218 | AAK1 |
| rs6734326 | 2:96903998-96903998 | FAM178B |
| rs144201388 | 2:107997366-107997366 | SLC5A7 |
| rs1219690 | 2:122487007-122487007 | AC011246.1 |
| rs78793148 | 2:136023692-136023692 | DARS-AS1 |
| rs77734975 | 2:144750626-144750626 | TEX41 |
| rs150409445 | 2:145016457-145016457 | TEX41 |
| rs723681 | 2:147910940-147910940 | ACVR2A |
| rs1965812 | 2:157814786-157814786 | ACVR1 |
| rs4547489 | 2:160302714-160302714 | RBMS1 |
| rs58757862 | 2:160378941-160378941 | RBMS1 |
| rs7572202 | 2:160429040-160429040 | RBMS1 |
| rs353128 | 2:165282098-165282098 | SCN2A |
| rs6740895 | 2:165283510-165283510 | SCN2A |
| rs353139 | 2:165289717-165289717 | SCN2A |
| rs1816918 | 2:165322847-165322847 | SCN2A |
| rs75629463 | 2:166127885-166127885 | SCN1A |
| rs7589835 | 2:166216341-166216341 | SCN9A |
| rs115032219 | 2:166265206-166265206 | SCN9A |
| rs141858735 | 2:179466193-179466193 | ZNF385B |
| rs262266 | 2:180012435-180012435 | - |
| rs58695784 | 2:180057221-180057221 | - |
| rs13397184 | 2:180961506-180961506 | - |
| rs4853505 | 2:190382596-190382596 | - |
| rs3811607 | 2:190435676-190435676 | MFSD6 |
| rs281787 | 2:199919515-199919515 | C2orf69 |
| rs28418905 | 2:208323118-208323118 | PIKFYVE |

| | | |
|---|---|---|
| rs145042764 | 2:208330711-208330711 | PIKFYVE |
| rs2271543 | 2:218277768-218277768 | PNKD |
| rs13000510 | 2:222500376-222500376 | SGPP2 |
| rs13010621 | 2:231009411-231009411 | SPATA3 |
| rs6758225 | 2:236030768-236030768 | AGAP1 |
| rs35449298 | 2:236034722-236034722 | AGAP1 |
| rs551859 | 2:238463195-238463195 | - |
| rs7353549 | 20:793346-793346 | - |
| rs2295545 | 20:3184040-3184040 | - |
| rs6037501 | 20:3210559-3210559 | ITPA |
| rs141459119 | 20:4623150-4623150 | RPS4XP2 |
| rs116158058 | 20:4652752-4652752 | RPS4XP2 |
| rs112864829 | 20:4861106-4861106 | SLC23A2 |
| rs4813918 | 20:10157279-10157279 | SNAP25-AS1 |
| rs11698183 | 20:25994949-25994949 | FAM182A |
| rs78184009 | 20:31851317-31851317 | DUSP15 |
| rs6119287 | 20:32815400-32815400 | MAPRE1 |
| rs293727 | 20:33345258-33345258 | BPIFB9P |
| rs6060042 | 20:34773918-34773918 | NCOA6 |
| rs6073118 | 20:43518146-43518146 | L3MBTL1 |
| rs729997 | 20:59411230-59411230 | - |
| rs1736023 | 21:15440563-15440563 | AJ009632.2 |
| rs12626383 | 21:15462709-15462709 | AJ009632.2 |
| rs1688101 | 21:19347156-19347156 | RNU1-139P |
| rs8131708 | 21:40047976-40047976 | DSCAM |
| rs9305690 | 21:40058461-40058461 | DSCAM |
| rs2837413 | 21:40067182-40067182 | DSCAM |
| rs10222092 | 21:40082331-40082331 | DSCAM |
| rs12482422 | 21:40605066-40605066 | DSCAM |
| rs56379273 | 21:44406603-44406603 | TRPM2 |
| rs5751062 | 22:41202049-41202049 | L3MBTL2 |
| rs9611522 | 22:41231920-41231920 | L3MBTL2 |
| rs7290404 | 22:41353792-41353792 | ZC3H7B |
| rs74667571 | 22:50348547-50348547 | PPP6R2 |
| rs17046610 | 3:7024398-7024398 | GRM7 |
| rs7652281 | 3:8764054-8764054 | OXTR |
| rs341839 | 3:21379929-21379929 | - |
| rs10764 | 3:33496538-33496538 | CLASP2 |
| rs6766294 | 3:33613646-33613646 | CLASP2 |
| rs17075283 | 3:43184728-43184728 | - |
| rs75228828 | 3:46071391-46071391 | - |
| rs1982510 | 3:47011447-47011447 | SETD2 |
| rs56273245 | 3:47273006-47273006 | KIF9 |
| rs34365302 | 3:52373396-52373396 | DNAH1 |
| rs613519 | 3:52434924-52434924 | SEMA3G |
| rs13083798 | 3:52615732-52615732 | PBRM1 |

| | | |
|---|---|---|
| rs6765687 | 3:52699090-52699090 | GLT8D1 |
| rs36051354 | 3:52951996-52951996 | SFMBT1 |
| rs2178563 | 3:54791743-54791743 | CACNA2D3 |
| rs114413724 | 3:56515937-56515937 | - |
| rs28404421 | 3:58306758-58306758 | RPP14 |
| rs13085519 | 3:63650920-63650920 | SNTN |
| rs139277004 | 3:85818345-85818345 | CADM2 |
| rs4928106 | 3:101044767-101044767 | - |
| rs62408931 | 3:137110076-137110076 | - |
| rs79329661 | 3:141569914-141569914 | RASA2 |
| rs7629065 | 3:152412201-152412201 | MBNL1 |
| rs2222275 | 3:155641090-155641090 | PLCH1 |
| rs115083552 | 3:156226229-156226229 | KCNAB1 |
| rs77931272 | 3:167967106-167967106 | - |
| rs1967027 | 3:176245762-176245762 | - |
| rs73191724 | 3:187271636-187271636 | MASP1 |
| rs116699996 | 5:28167023-28167023 | - |
| rs3797237 | 5:54241912-54241912 | ARL15 |
| rs6453214 | 5:76288598-76288598 | SV2C |
| rs10060273 | 5:80322830-80322830 | SPZ1 |
| rs112216323 | 5:80352963-80352963 | AC026410.3 |
| rs13161403 | 5:88471144-88471144 | AC091826.3 |
| rs60470187 | 5:90756146-90756146 | ADGRV1 |
| rs76801032 | 5:107836324-107836324 | - |
| rs28786120 | 5:120359407-120359407 | AC113418.1 |
| rs112723361 | 5:120490287-120490287 | PRR16 |
| rs4282308 | 5:124690450-124690450 | ZNF608 |
| rs11241766 | 5:124741569-124741569 | ZNF608 |
| rs6875262 | 5:124774580-124774580 | - |
| rs1078675 | 5:124881569-124881569 | LINC02240 |
| rs57750646 | 5:132344571-132344571 | SLC22A4 |
| rs13361619 | 5:138312990-138312990 | CDC25C |
| rs3798153 | 5:138446515-138446515 | REEP2 |
| rs12657476 | 5:151186635-151186635 | CCDC69 |
| rs113516381 | 5:177030476-177030476 | ZNF346 |
| rs3846912 | 6:13656808-13656808 | RANBP9 |
| rs1204148 | 6:13817127-13817127 | MCUR1 |
| rs625964 | 6:20016736-20016736 | - |
| rs6909187 | 6:25785697-25785697 | SLC17A1 |
| rs62396221 | 6:26285520-26285520 | H4C8 |
| rs9378107 | 6:28793906-28793906 | - |
| rs1265075 | 6:31145336-31145336 | CCHCR1 |
| rs9263850 | 6:31188411-31188411 | - |
| rs28397271 | 6:31196866-31196866 | HCG27 |
| rs6930643 | 6:31206258-31206258 | HCG27 |
| rs28744244 | 6:31209999-31209999 | - |

| | | |
|---|---|---|
| rs2853923 | 6:31297960-31297960 | LINC02571 |
| rs6909636 | 6:31378822-31378822 | ZDHHC20P2 |
| rs3130062 | 6:31558135-31558135 | NFKBIL1 |
| rs2857609 | 6:31610048-31610048 | UQCRHP1 |
| rs1442222 | 6:46246324-46246324 | RCAN2 |
| rs9389810 | 6:99881570-99881570 | - |
| rs36142021 | 6:118838319-118838319 | MCM9 |
| rs12206988 | 6:119085363-119085363 | FAM184A |
| rs6569038 | 6:119095307-119095307 | FAM184A |
| rs7769687 | 6:125912545-125912545 | NCOA7 |
| rs12662170 | 6:150925255-150925255 | MTHFD1L |
| rs9365002 | 6:158903689-158903689 | C6orf99 |
| rs77846331 | 6:159910464-159910464 | MAS1 |
| rs7744123 | 6:168219979-168219979 | AL606970.5 |
| rs73047368 | 7:2726762-2726762 | GNA12 |
| rs78391336 | 7:2793074-2793074 | GNA12 |
| rs113918424 | 7:6333933-6333933 | FAM220A |
| rs61247228 | 7:6612156-6612156 | C7orf26 |
| rs12702597 | 7:7272864-7272864 | AC005532.1 |
| rs140622649 | 7:36358639-36358639 | KIAA0895 |
| rs78241966 | 7:36446251-36446251 | ANLN |
| rs41279603 | 7:43943193-43943193 | UBE2D4 |
| rs62469301 | 7:65202049-65202049 | INTS4P1 |
| rs1075007 | 7:78481252-78481252 | MAGI2 |
| rs62461391 | 7:82854172-82854172 | PCLO |
| rs76083843 | 7:89304380-89304380 | ZNF804B |
| rs10215799 | 7:94675032-94675032 | - |
| rs17166875 | 7:95411959-95411959 | PON2 |
| rs2237585 | 7:95420506-95420506 | PON2 |
| rs4595055 | 7:95707291-95707291 | - |
| rs6979335 | 7:100492237-100492237 | NYAP1 |
| rs7805498 | 7:100537297-100537297 | AGFG2 |
| rs4599742 | 7:106050650-106050650 | - |
| rs76305012 | 7:117557301-117557301 | CFTR |
| rs79006095 | 7:120333784-120333784 | KCND2 |
| rs73166011 | 7:149436878-149436878 | ZNF777 |
| rs1971411 | 8:8415897-8415897 | AC103957.1 |
| rs2921073 | 8:8450133-8450133 | - |
| rs1532278 | 8:27608798-27608798 | CLU |
| rs7837112 | 8:28907976-28907976 | HMBOX1 |
| rs7011522 | 8:35029242-35029242 | - |
| rs118027082 | 8:73952340-73952340 | ELOC |
| rs117582595 | 8:104624038-104624038 | ZFPM2 |
| rs12541194 | 8:104637830-104637830 | ZFPM2 |
| rs6469271 | 8:109632545-109632545 | SYBU |
| rs800902 | 8:115416489-115416489 | TRPS1 |

| | | |
|---|---|---|
| rs73337990 | 8:124422412-124422412 | - |
| rs17372350 | 8:124576693-124576693 | MTSS1 |
| rs3857948 | 8:124598539-124598539 | MTSS1 |
| rs75393804 | 8:134506857-134506857 | ZFAT |
| rs75582926 | 8:134523293-134523293 | ZFAT |
| rs1060832 | 8:140531789-140531789 | AGO2 |
| rs149865921 | 8:141499890-141499890 | MROH5 |
| rs117511259 | 9:4064097-4064097 | GLIS3 |
| rs78062691 | 9:32257417-32257417 | - |
| rs76639411 | 9:80868137-80868137 | AL138749.1 |
| rs2025943 | 9:89153960-89153960 | SHC3 |
| rs7862334 | 9:90493810-90493810 | LINC01501 |
| rs10821079 | 9:93161958-93161958 | - |
| rs13289468 | 9:125418053-125418053 | AL627223.2 |
| rs13283793 | 9:125679362-125679362 | MAPKAP1 |
| rs113253813 | 9:127498834-127498834 | LRSAM1 |

**Supplementary table 5: Counts of incident PD cases and controls within each decile of the PRS in the testing (validation) cohort.**

| Decile (1=lowest risk, 10= highest risk) | Controls | Cases | Case prevalence within decile |
|---|---|---|---|
| 1 | 21651 | 28 | 0.001291572 |
| 2 | 21635 | 38 | 0.001753334 |
| 3 | 21616 | 52 | 0.002399852 |
| 4 | 21623 | 51 | 0.00235305 |
| 5 | 21604 | 57 | 0.002631457 |
| 6 | 21612 | 53 | 0.002446342 |
| 7 | 21598 | 59 | 0.002724292 |
| 8 | 21588 | 57 | 0.002633403 |
| 9 | 21559 | 74 | 0.0034207 |
| 10 | 21543 | 82 | 0.003791908 |

**Supplementary table 6: Reproduced with permission from Nalls et al 2019. PD risk loci from the Meta5 GWAS. Columns show RSID and nearest genes. To determine how much variance explained by the PRS was due to previously undiscovered loci, all variants within 1MB of these loci were excluded.**

| SNP | Nearest Gene |
|---|---|
| rs114138760 | PMVK |
| rs35749011 | KRTCAP2 |
| rs76763715 | GBAP1 |
| rs6658353 | FCGR2A |
| rs11578699 | VAMP4 |
| rs823118 | NUCKS1 |
| rs11557080 | RAB29 |
| rs4653767 | ITPKB |
| rs10797576 | SIPA1L2 |
| rs76116224 | KCNS3 |
| rs2042477 | KCNIP3 |
| rs11683001 | MAP4K4 |
| rs57891859 | TMEM163 |
| rs1474055 | STK39 |
| rs73038319 | SATB1 |
| rs6808178 | LINC00693 |
| rs12497850 | IP6K2 |
| rs55961674 | KPNA1 |
| rs11707416 | MED12L |
| rs1450522 | SPTSSB |
| rs10513789 | MCCC1 |
| rs873786 | GAK |
| rs34311866 | TMEM175 |
| rs4698412 | BST1 |
| rs34025766 | LCORL |
| rs6825004 | SCARB2 |
| rs4101061 | FAM47E |
| rs6854006 | FAM47E-STBD1 |
| rs356182 | SNCA |
| rs5019538 | SNCA |
| rs13117519 | CAMK2D |
| rs62333164 | CLCN3 |
| rs1867598 | ELOVL7 |
| rs26431 | PAM |
| rs11950533 | C5orf24 |
| rs4140646 | LOC100131289 |
| rs9261484 | TRIM40 |
| rs112485576 | HLA-DRB5 |

| | |
|---|---|
| rs12528068 | RIMS1 |
| rs997368 | FYN |
| rs75859381 | RPS12 |
| rs199351 | GPNMB |
| rs76949143 | GS1-124K5.11 |
| rs1293298 | CTSB |
| rs620513 | FGF20 |
| rs2280104 | BIN3 |
| rs2086641 | FAM49B |
| rs13294100 | SH3GL2 |
| rs10756907 | SH3GL2 |
| rs6476434 | UBAP2 |
| rs896435 | ITGA8 |
| rs10748818 | GBF1 |
| rs72840788 | BAG3 |
| rs117896735 | INPP5F |
| rs7938782 | RNF141 |
| rs12283611 | DLG2 |
| rs3802920 | IGSF9B |
| rs76904798 | LRRK2 |
| rs34637584 | LRRK2 |
| rs7134559 | SCAF11 |
| rs10847864 | HIP1R |
| rs11610045 | FBRSL1 |
| rs9568188 | CAB39L |
| rs4771268 | MBNL2 |
| rs12147950 | MIPOL1 |
| rs11158026 | GCH1 |
| rs3742785 | RPS6KL1 |
| rs979812 | GALC |
| rs2251086 | VPS13C |
| rs6497339 | SYT17 |
| rs2904880 | CD19 |
| rs11150601 | SETD1A |
| rs6500328 | NOD2 |
| rs3104783 | CASC16 |
| rs10221156 | CHD9 |
| rs12600861 | CHRNB1 |
| rs12951632 | RETREG3 |
| rs2269906 | UBTF |
| rs850738 | FAM171A2 |
| rs62053943 | CRHR1 |

| | |
|---|---|
| rs117615688 | CRHR1 |
| rs11658976 | WNT3 |
| rs61169879 | BRIP1 |
| rs666463 | DNAH17 |
| rs1941685 | ASXL3 |
| rs12456492 | RIT2 |
| rs8087969 | MEX3C |
| rs55818311 | SPPL2B |
| rs77351827 | CRLS1 |
| rs2248244 | DYRK1A |

**Supplementary table 7: multiplicative interaction term betas and associated P values for traits associated with PD x polygenic risk score. Interaction terms are derived from logistic regression models of the form PD status ~ Age + Sex + first four genetic PCs + risk factor + PRS + PRS x risk factor. P values are derived from likelihood ratio tests. The estimate for menarche is derived from a model without a sex term as it was restricted to females.**

| Exposure | Beta | P.value | FDR |
|---|---|---|---|
| Diabetes | -0.403295781 | 0.026329263 | 0.263292625 |
| PD FHx | 0.200189481 | 0.173728893 | 0.424421204 |
| Sleepiness | -0.130869523 | 0.151841837 | 0.424421204 |
| Dementia FHx status | -0.138276444 | 0.212210602 | 0.424421204 |
| Menarche | 0.060885645 | 0.184540506 | 0.424421204 |
| Epilepsy | -0.27737041 | 0.305717602 | 0.509529336 |
| Depression | -0.108061776 | 0.483702106 | 0.642356797 |
| Smoking status | 0.047715657 | 0.578121118 | 0.642356797 |
| Gastric Ulcer | -0.156191177 | 0.557395514 | 0.642356797 |
| Alcohol | 0.022188797 | 0.808528932 | 0.808528932 |
