# Supplementary material for: Parkinson’s disease determinants, prediction and gene–environment interactions in the UK Biobank
Source: J Neurol Neurosurg Psychiatry. 2020 Sep 14;91(10):1046–54. doi: 10.1136/jnnp-2020-323646 (PMC7509524; doi:10.1136/jnnp-2020-323646)

Supplementary figure 1: Sensitivity analysis for incident case-control study. For this analysis only individuals of self-declared 'White' ethnicity were included. Associations of risk factors and incident cases of Parkinson's disease. Point estimates for association are depicted as log odds ratios and 95% confidence intervals. Estimates of association were derived from logistic regression models adjusting for age, sex, Townsend deprivation index at recruitment, and ethnicity. BMI = body mass index; PD = Parkinson's disease.

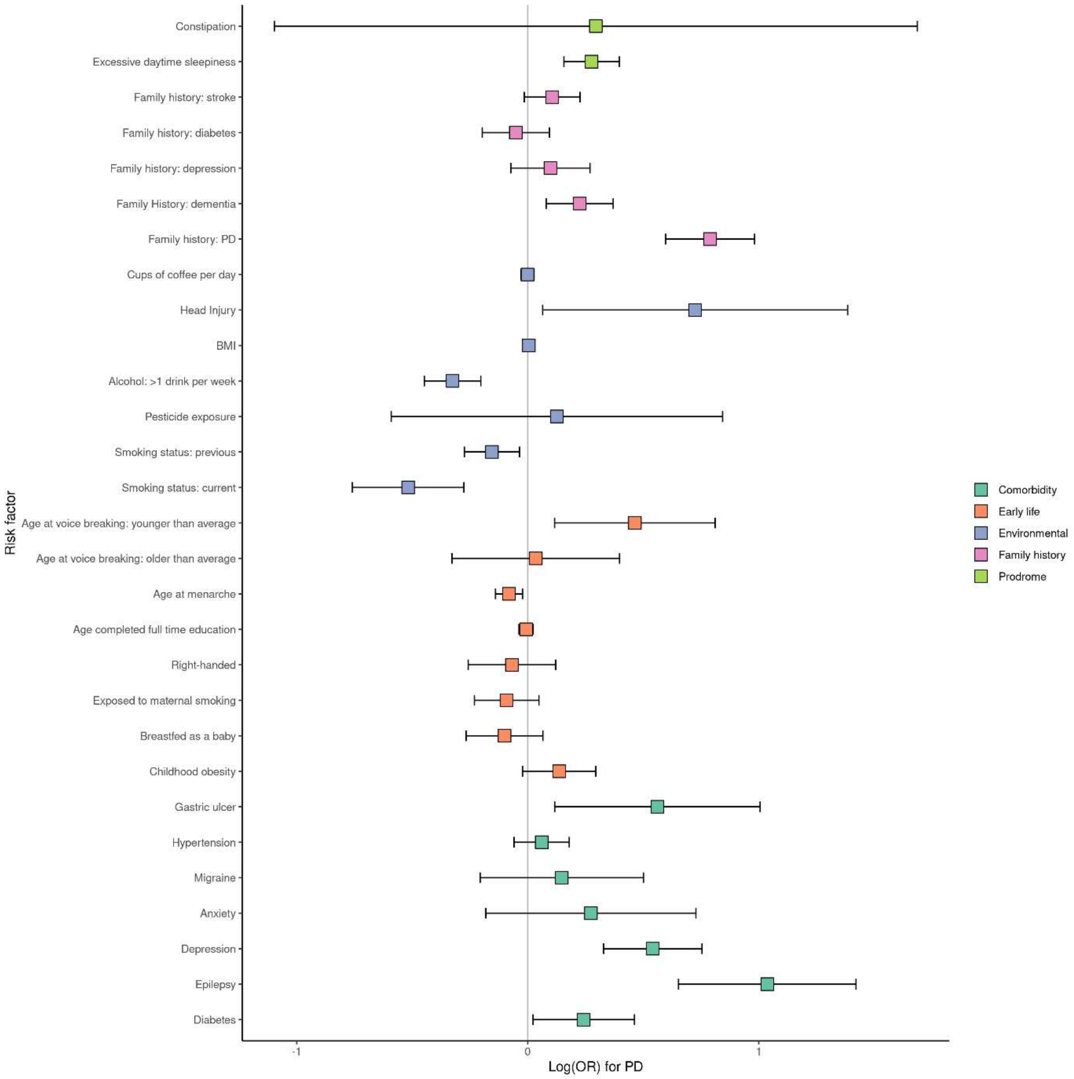

Supplementary figure 2: Sensitivity analysis for incident case-control study. For this analysis only individuals above the age of 60 at recruitment were included. Associations of risk factors and incident cases of Parkinson's disease. Point estimates for association are depicted as log odds ratios and 95% confidence intervals. Estimates of association were derived from logistic regression models adjusting for age, sex, Townsend deprivation index at recruitment, and ethnicity. BMI = body mass index; PD = Parkinson's disease.

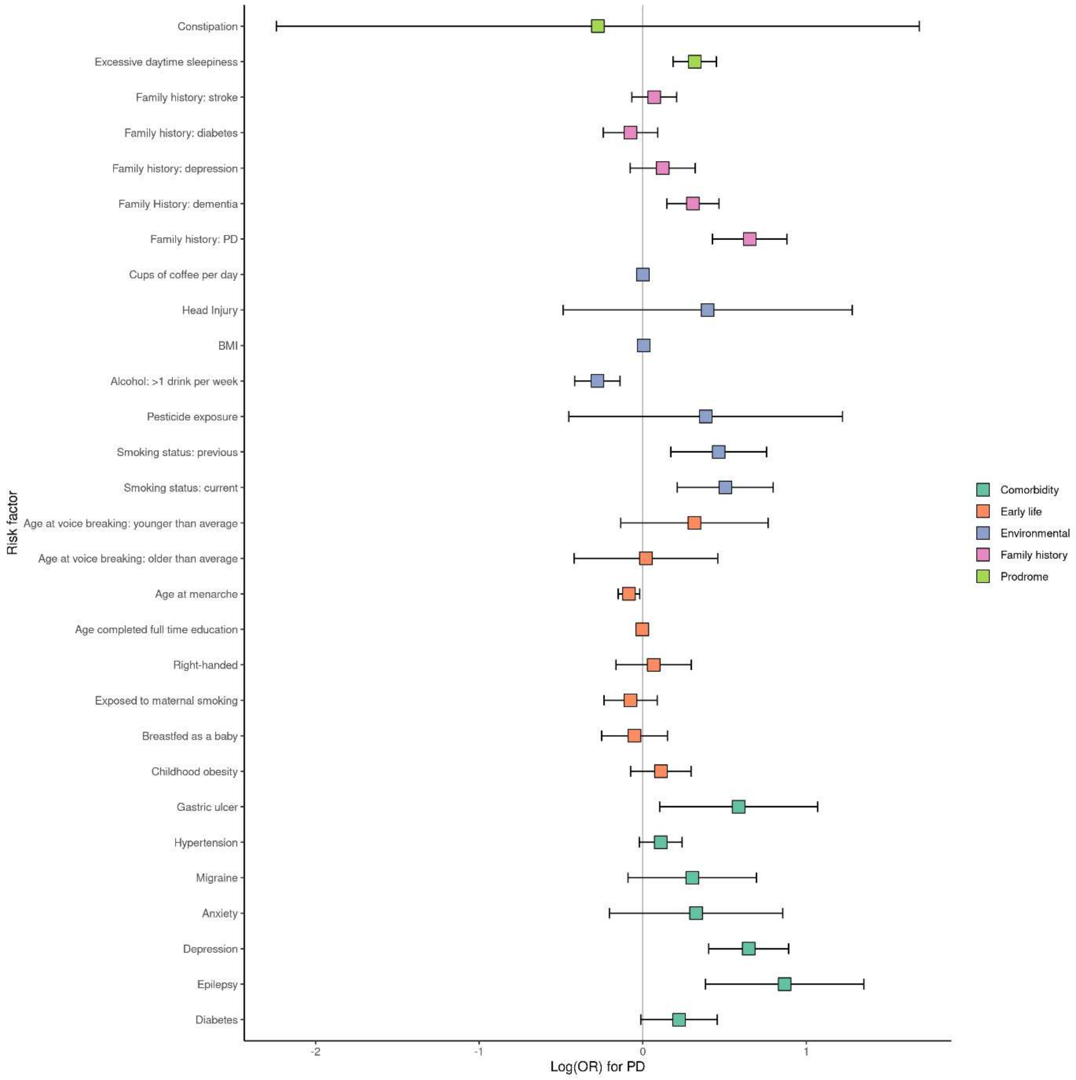

Supplementary figure 3: Sensitivity analysis for incident case-control study. For this analysis, we included all incident PD cases, but used matched controls rather than the entire cohort. Controls were matched 4:1 for age at recruitment, sex, and ethnicity (white vs non-white). Associations of risk factors and incident cases of Parkinson's disease. Point estimates for association are depicted as log odds ratios and 95% confidence intervals. Estimates of association were derived from logistic regression models adjusting for age, sex, Townsend deprivation index at recruitment, and ethnicity. BMI = body mass index; PD = Parkinson's disease.

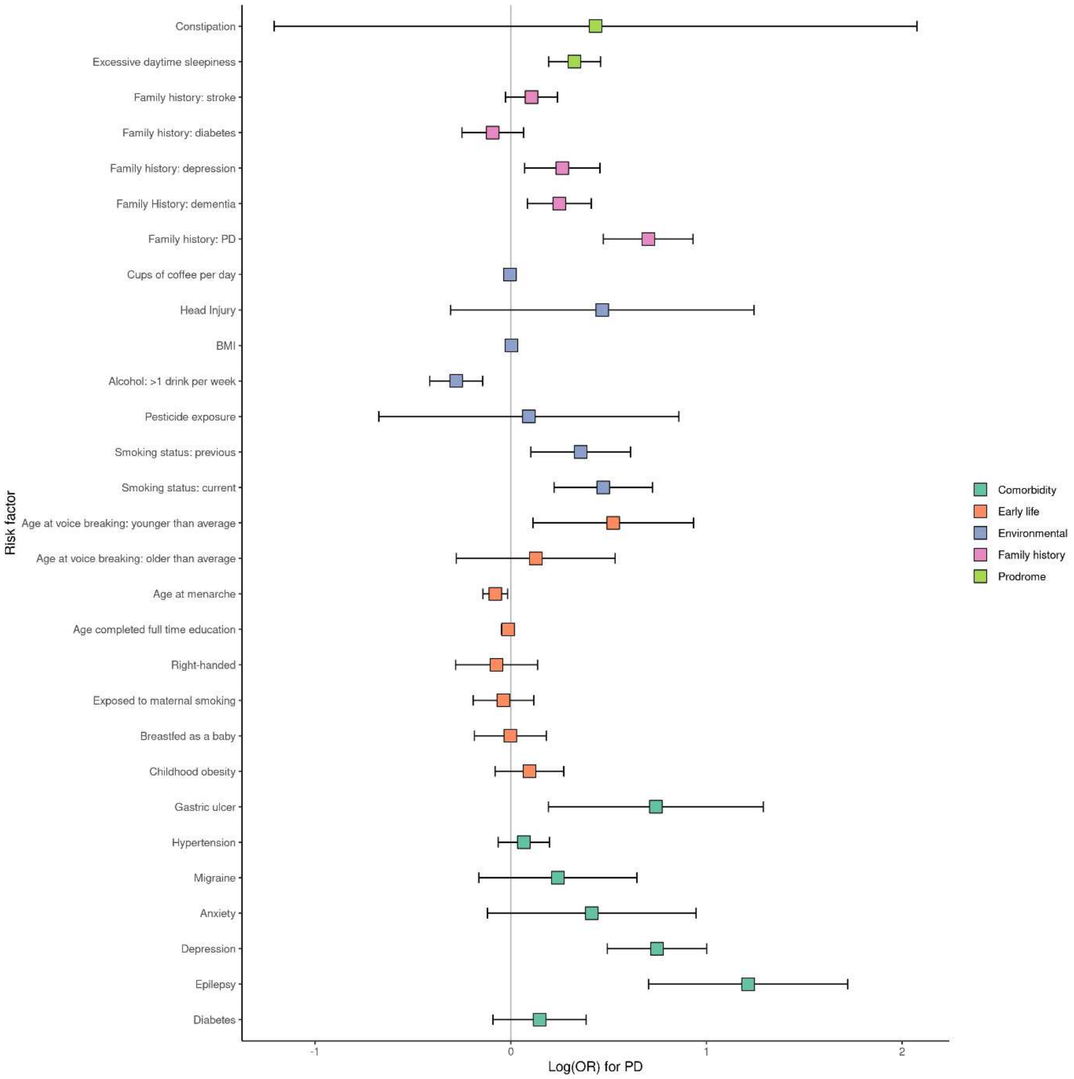

Supplementary figure 4: Sensitivity analysis for incident case-control study. For this analysis, we exclude incident PD cases whose sole source of PD diagnosis on record was self-report (33 cases excluded). Associations of risk factors and incident cases of Parkinson's disease. Point estimates for association are depicted as log odds ratios and 95% confidence intervals. Estimates of association were derived from logistic regression models adjusting for age, sex, Townsend deprivation index at recruitment, and ethnicity. BMI = body mass index; PD = Parkinson's disease.

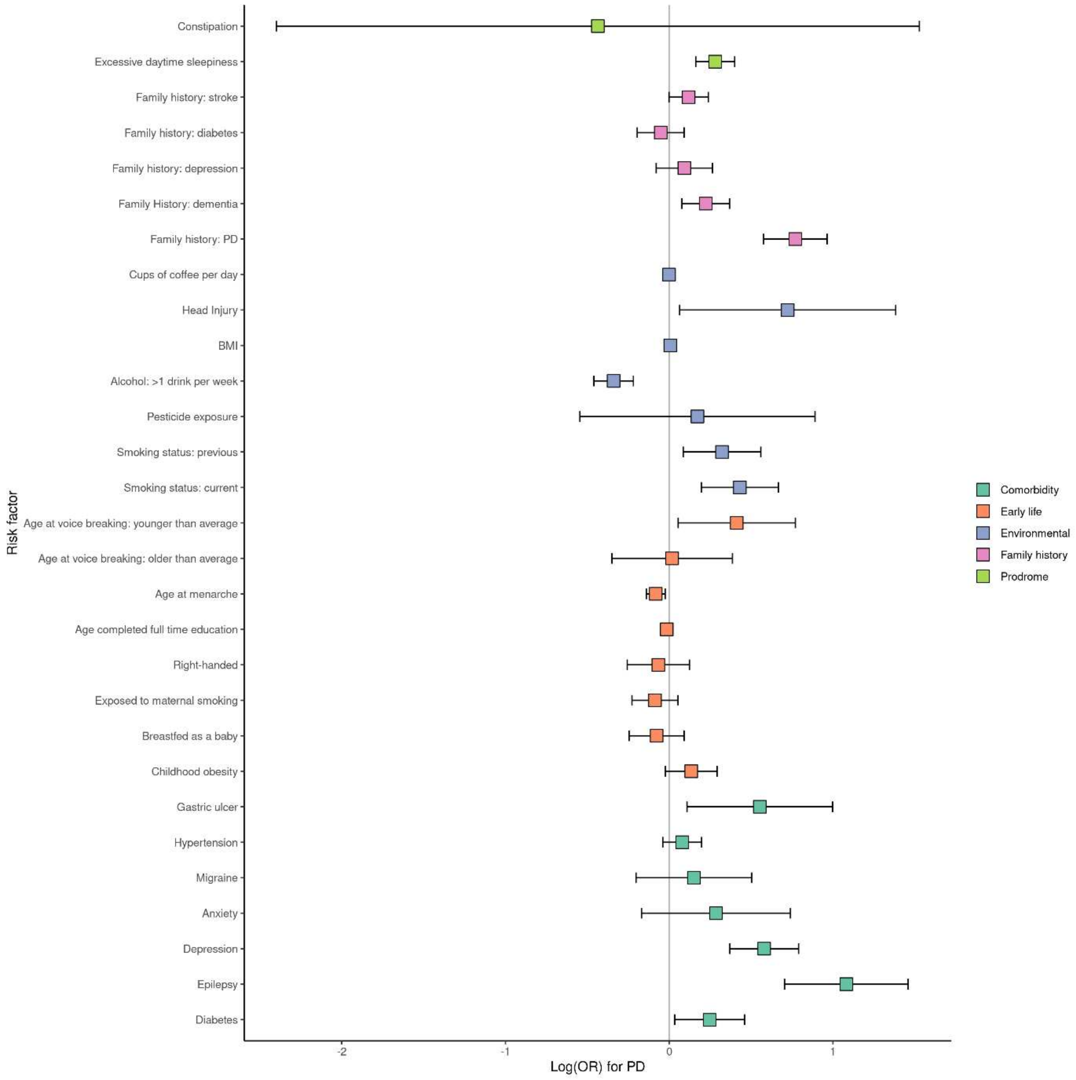

Supplementary figure 5: Calibration plot depicting absolute PD risk on the observed scale (no. of cases/total no. of participants) within each decile, and predicted PD risk from various models. The models assessed were as follows: PREDICT-PD + genetic PCs, genetic PCs alone, PREDICT-PD + genetic PCs + PRS, and genetic PCs + PRS. Predicted probabilities were determined from multivariable logistic regression models, modelling incident PD risk on the included covariates. The PRS was transformed using the inverse normal transformation. PREDICT-PD estimates of baseline risk were included in the model as the predicted log odds of PD. The first 4 genetic PCs were included. Inclusion of the PRS improves calibration within all deciles compared to null models including only genetic PCs and the PREDICT-PD algorithm.

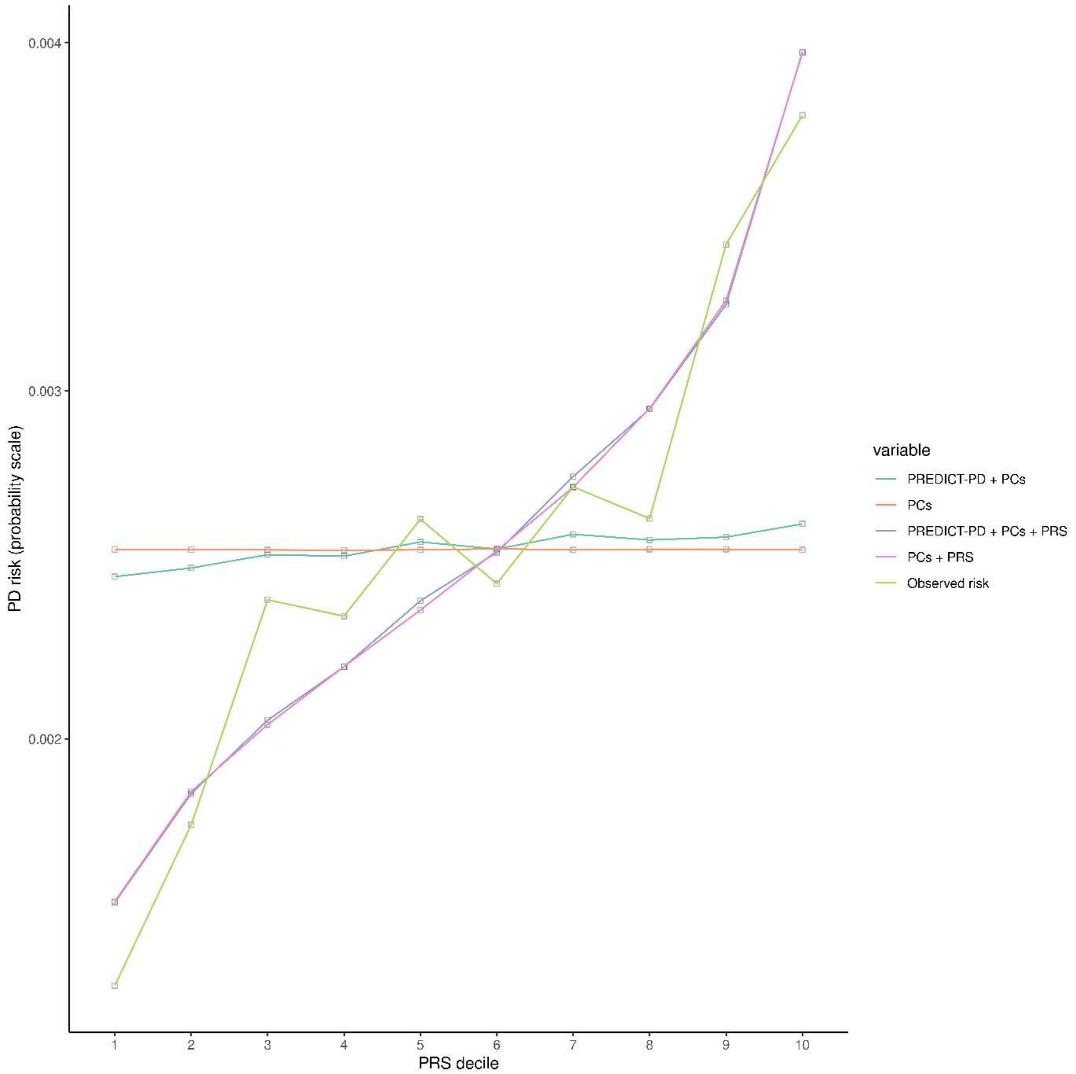

Supplementary figure 6: Receiver Operating Characteristic (ROC) curves depicting the discriminative capacity of various models for distinguishing incident PD cases from controls. The models assessed were as follows: PREDICT-PD + genetic PCs, genetic PCs alone, PREDICT-PD + genetic PCs + PRS, and genetic PCs + PRS. Predicted probabilities were determined from multivariable logistic regression models, modelling incident PD risk on the included covariates. The PRS was transformed using the inverse normal transformation. PREDICT-PD estimates of baseline risk were included in the model as the predicted log odds of PD. The first 4 genetic PCs were included. Inclusion of the PRS improves calibration within all deciles compared to null models including only genetic PCs and the PREDICT-PD algorithm.

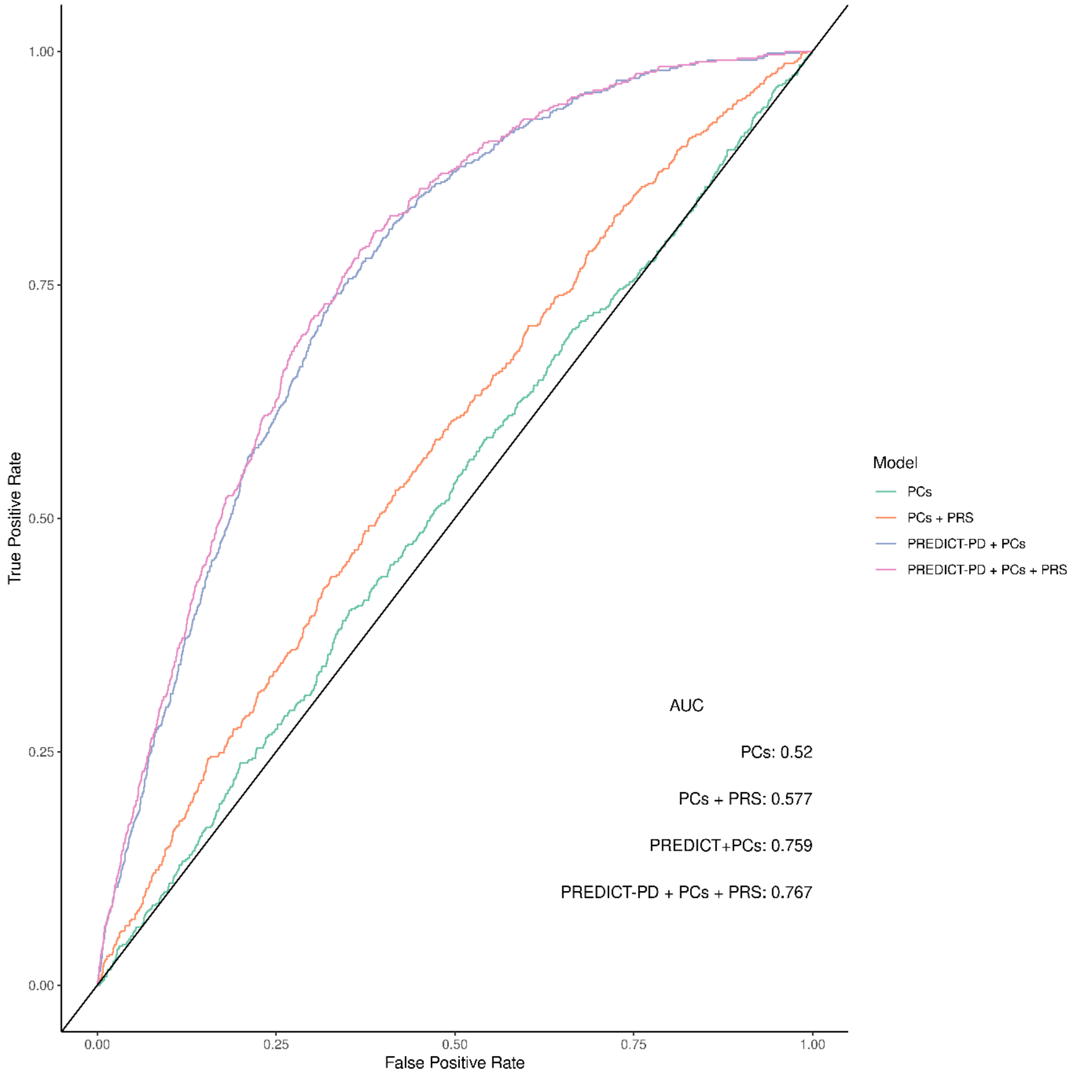

Supplementary figure 7: Genetic principal components (PC) plot of all participants with genetic data prior to exclusion of related and non-European individuals. PCs were supplied by UK Biobank. Participants are labelled by self-reported ethnic background.

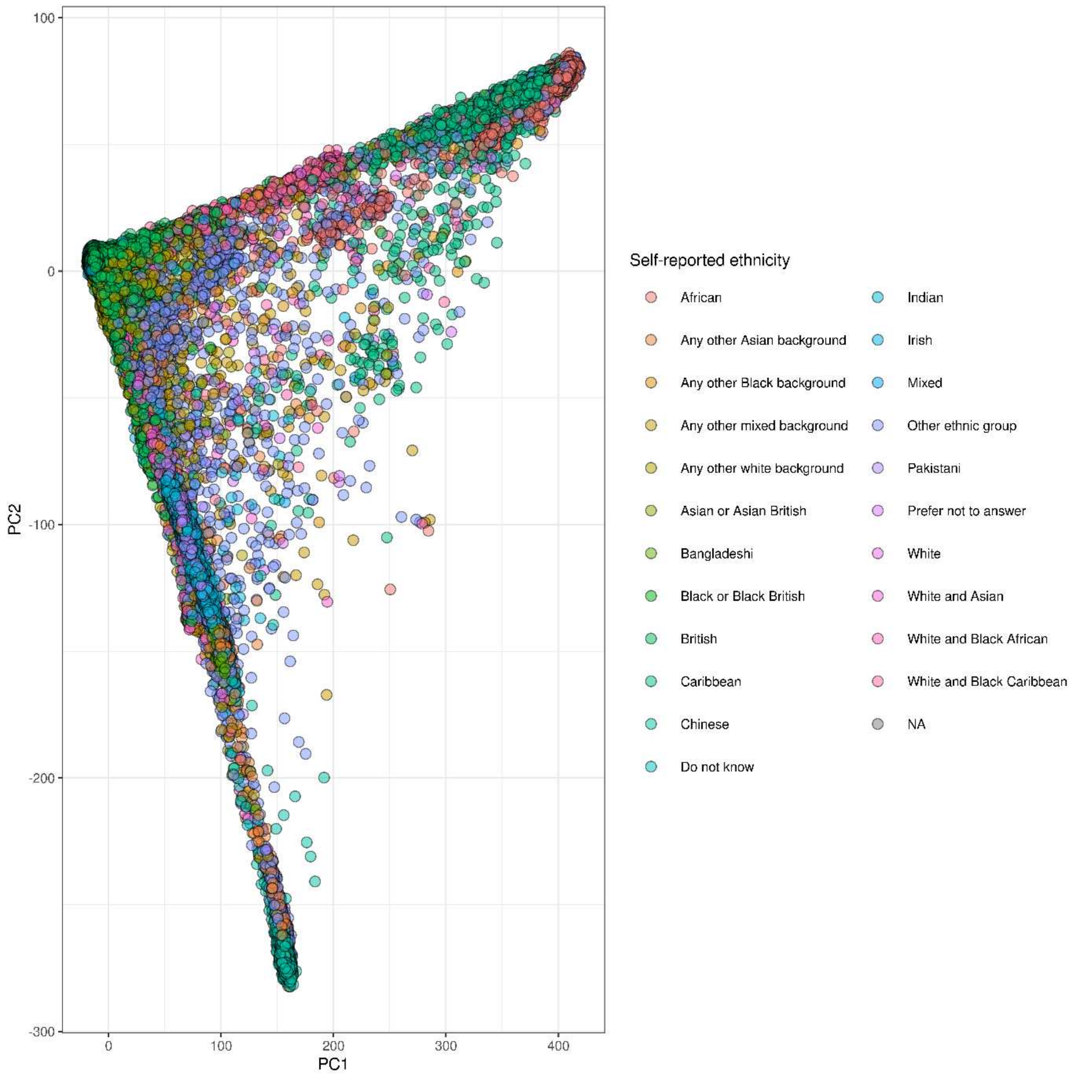

Supplementary figure 8: Genetic principal components (PC) plot of all participants with genetic data after exclusion of related and non-European individuals. PCs were supplied by UK Biobank. Participants are labelled by self-reported ethnic background.

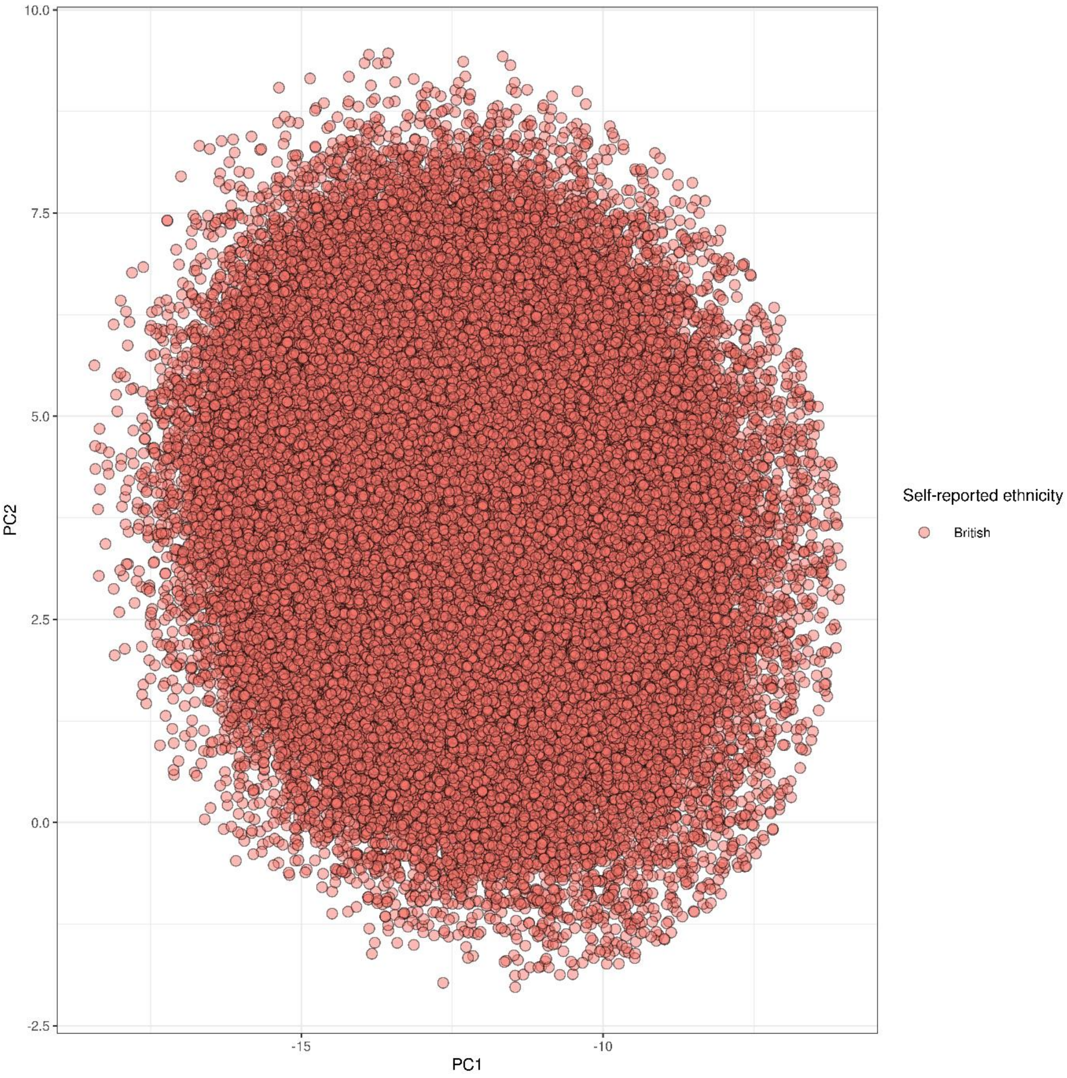

Supplement: Supplementary data [file jnnp-2020-323646supp002.pdf]
